# Health-Related Quality-of-Life Assessment in Dementia: Evidence of Cross-Cultural Validity in Latin America

**DOI:** 10.1037/pas0000743

**Published:** 2019-07-08

**Authors:** Kia-Chong Chua, Jan R. Böhnke, Martin Prince, Sube Banerjee

**Affiliations:** 1 Centre for Implementation Science, Institute of Psychiatry, Psychology, and Neuroscience, King’s College London, and Quality Improvement and SLaM Partners, South London and Maudsley NHS Foundation Trust, London, United Kingdom; 2 School of Nursing and Health Sciences, University of Dundee; 3 King’s Global Health Institute, Institute of Psychiatry, Psychology, and Neuroscience, King’s College London; 4 Centre for Dementia Studies, Brighton and Sussex Medical School, University of Sussex

**Keywords:** dementia, health-related quality-of-life, cross-cultural validation, measurement invariance

## Abstract

All health-related quality-of-life (HRQL) measures for dementia have been developed in high-income countries and none were validated for cross-cultural use. Yet, the global majority of people living with dementia reside in low- and middle-income countries. We therefore investigated the measurement invariance of a set of self- and informant-report HRQL measures developed in the United Kingdom when used in Latin America. Self-reported HRQL was obtained using (DEMQOL) at a memory assessment service in the United Kingdom (*n* = 868) and a population cohort study in Latin America (*n* = 417). Informant reports were collected using DEMQOL-Proxy at both sites (*n* = 909 and *n* = 495). Multiple-group confirmatory bifactor models for ordered categorical item responses were estimated to evaluate measurement invariance. Results support configural, metric, and scalar invariance for the concept of general HRQL in DEMQOL and DEMQOL-Proxy. The dominant impact of general HRQL on item responses was evident across U.K. English and Ibero American Spanish versions of DEMQOL (ω_*h*_ = 0.87–0.90) and DEMQOL-Proxy (ω_*h*_ = 0.88–0.89). Ratings of “positive emotion” did not show a major impact on general HRQL appraisal, particularly for Latin American respondents. Item information curves show that self- and informant-reports were highly informative about the presence rather than the absence of HRQL impairment. We found no major difference in conceptual meaning, sensitivity, and relevance of DEMQOL and DEMQOL-Proxy for older adults in the United Kingdom and Latin America. Further replication is needed for consensus over which HRQL measures are appropriate for making cross-national comparisons in global dementia research.

## Public Significance Statement

Measuring health-related quality-of-life (HRQL) in older adults living with dementia is important to quantify their burden of disease. Globally more than half of these adults live in low- and middle-income countries, but all HRQL measures for dementia originate from high-income countries. Our study is the first to show for a self- and informant-report HRQL instrument (DEMQOL) that the appraisals are comparable across U.K. (English) and Latin American (Spanish) community samples.

According to the 2010 World Alzheimer Report (Wimo & Prince, 2010), the likelihood of developing dementia roughly doubles every 5 years after the age of 65. As the number of people reaching the age of 65 is growing rapidly due to population ageing worldwide, the number of people living with dementia is expected to rise globally (Prince, Guerchet, & Prina, 2015).

Dementia brings about a decline in memory, reasoning, and communication skills, and a gradual loss of skills needed in daily life for independent living (Knapp et al., 2007). At any stage of illness, individuals may also develop behavioral and psychological symptoms of dementia such as depression, psychosis (hallucinations and delusions), aggression, and wandering. Available drug treatment may improve symptoms temporarily, but none has been shown to slow or stop the disease process (The Alzheimer’s Association, 2013). Current standard treatments continue to be the subject of clinical trials due to long-standing concerns over drug efficacy and safety (Ballard et al., 2009; Banerjee et al., 2011). With prevailing challenges in the treatment of dementia, the goal of “adding years to life” also needs to consider the goal of “adding life to years” (Clark, 1995).

Cognitive functioning is fundamentally a core outcome of disease-modifying treatment in dementia (Webster et al., 2017). However, interventions whose efficacy is tested on change in standardized cognitive test performance may not capture outcomes of greatest relevance to the lived experience of people with dementia (Harrison, Noel-Storr, Demeyere, Reynish, & Quinn, 2016). The goal of “adding life to years” needs an examination of dementia’s impact on the whole person. This is the purpose of health-related quality-of-life (HRQL) measures (Dichter, Schwab, Meyer, Bartholomeyczik, & Halek, 2016).

Despite the clarity of purpose, the absence of a theoretical framework unique for HRQL in dementia has resulted in the emergence of at least 18 measures over the past 20 years (Missotten, Dupuis, & Adam, 2016). This diversity prompted calls for consensus over what should be the standard measurement tool for HRQL in dementia. Under the Core Outcome Measures in Effectiveness Trials initiative, efforts to establish evidence-based consensus on measurement tools have focused on community care settings for people with dementia (Reilly et al., 2014), as well as for disease modification trials in mild-to-moderate dementia (Webster et al., 2017).

A key motive for encouraging use of a standard HRQL measure across evaluation purposes is the need for making direct comparison between different types of dementia care interventions that incur disparate amount of resources to improve psychosocial outcomes (e.g., Cooper et al., 2012; Knapp, Iemmi, & Romeo, 2013; Spijker et al., 2008). Common use of a standard HRQL measure can also enhance interprofessional communication in clinical care (Bentvelzen, Aerts, Seeher, Wesson, & Brodaty, 2017). However, even with standard outcome measures in dementia like the Mini-Mental State Examination (MMSE), factors like ethnicity can distort measurements when there are no genuine differences (Dai et al., 2013; Jones, 2006). The use of a standard HRQL measure with diverse population needs investigation of such measurement issues.

In disease modification trials, where HRQL is increasingly considered for secondary outcome monitoring (Harrison et al., 2016), the need for meta-analyses to determine the overall benefit of treatment regimens is likely to involve comparisons of multiple clinical trials from different countries (e.g., Perng, Chang, & Tzang, 2018). Here, potential measurement issues due to the use of a standard HRQL measure with diverse populations may be accentuated in such international comparisons.

With increasing application across broader settings in diverse cultures, HRQL measurement in dementia faces a uniquely urgent challenge. From 2015 to 2050, the number of people living with dementia is predicted to increase about twofold in Europe and North America, threefold in Asia, and fourfold in Latin America and Africa (Prince, Guerchet, et al., 2015). More than half the world population of people with dementia currently live in regions classified by the World Bank as low- and middle-income countries and by 2050 this proportion is expected to rise to close to 70% due to population ageing (Prince, Wimo, et al., 2015). Although the global majority of people living with dementia resides in low- and middle-income countries, all HRQL measures for dementia have been developed in high-income countries and none is sufficiently validated to support use in cultures other than that where the original development took place (Dichter et al., 2016).

To date, among dementia-specific HRQL measures, the DEMQOL system (Mulhern et al., 2013) has the best evidence of responsiveness to minimum clinically important difference in cognitive function, behavioral and psychological symptoms in dementia, functioning in activities of daily living, and depression (Bentvelzen et al., 2017). Recent reviews have also consistently identified the DEMQOL system as among the most commonly used HRQL measures for dementia intervention and disease-modifying trials (Harrison et al., 2016; Webster et al., 2017). With self- and informant-report versions (DEMQOL and DEMQOL-Proxy), the DEMQOL system also highlight the need for comparing both perspectives to determine the utility of proxy report, especially for later stages of illness when self-report is often not available.

The prospect of standardizing HRQL measurement in dementia will need attention on measurement validity across cultures (Prince, 2008). We therefore used a unique dataset of HRQL assessments using DEMQOL and DEMQOL-Proxy, dementia-specific HRQL measures developed in the United Kingdom (Mulhern et al., 2013), to evaluate and cross-culturally validate its use in the United Kingdom, the Dominican Republic, Mexico, Cuba, Peru, and Venezuela.

## Method

### Study Participants

We conducted secondary data analysis on completely deidentified data drawn from two primary studies that had obtained ethics approval (Banerjee et al., 2007; Prince et al., 2007). The first comprised community-dwelling older adults attending a London memory assessment service. This is a diagnostic service that focuses on early diagnosis and intervention. Referrals to the team are made from primary care and a clinical diagnosis of dementia is made following a comprehensive multidisciplinary assessment including self- and informant-report HRQL using DEMQOL and DEMQOL-Proxy (Banerjee et al., 2007).

The second comprised community-dwelling older adults with dementia identified in population cohort surveys conducted by the 10/66 Dementia Research Group in the Dominican Republic, Mexico, Cuba, Peru and Venezuela. Self- and informant-report HRQL were obtained in the first follow up (2007–2010) for participants identified in the baseline survey (2003–2007) to have dementia based on a battery of interview assessments (Rodriguez et al., 2008).

The study participants in both the U.K. and Latin American samples had a similar age range (see Table 1). Over half were female (63–72%). The majority (85–97%) in both study samples had mild to moderately severe dementia.[Table-anchor tbl1]

### Measures

DEMQOL (28 items) and DEMQOL-Proxy (31 items) are interviewer-administered measures for obtaining self- and informant-reports on the HRQL of people with dementia. Items inquire about “feelings,” “memory,” and “everyday life” of the person with dementia in the last week, with four responses ranging from 1 (*a lot*) to 4 (*not at all*). Reverse scoring is required for five “positive emotion” items in DEMQOL and DEMQOL-Proxy so that higher total scores reflect better HRQL. Studies reported evidence of responsiveness, convergent validity, structural validity, and measurement reliability (Chua et al., 2016; Mulhern et al., 2013).

As part of their research program on dementia in low- and middle-income countries, the 10/66 Dementia Research Group adapted DEMQOL and DEMQOL-Proxy through forward and back-translation and administered Ibero American Spanish versions to measure HRQL in community-dwelling older adults living with dementia in the Dominican Republic, Mexico, Cuba, Peru, and Venezuela (Prina et al., 2017).

Dementia severity was summarized using the MMSE (Folstein, Folstein, & McHugh, 1975) for the U.K. sample, and the Clinical Dementia Rating (CDR; C. P. Hughes, Berg, Danziger, Coben, & Martin, 1982) for respondents in Latin America. The MMSE is a screening tool for general cognitive impairment, with higher total scores (range = 0–30) indicating better performance, and studies have reported evidence of structural validity (Rubright, Nandakumar, & Karlawish, 2016), predictive validity, and reliability (Tombaugh & McIntyre, 1992). The CDR is a standardized semistructured interview with self- and informant inputs on cognitive and functional performance. The scale ratings (0–3) have shown evidence of criterion-validity, interrater reliability and have been validated neuropathologically for dementia (Morris, 1997). Substantial agreement between the MMSE and CDR has been documented for mild (κ = 0.62), moderate (κ = 0.69), and severe dementia (κ = 0.76; Perneczky et al., 2006).

### Statistical Analysis

For cross-cultural validity, DEMQOL and DEMQOL-Proxy need to retain the same conceptual meaning, sensitivity and relevance (“measurement invariance”) after they have been translated from English to Ibero American Spanish. An initial careful process of forward and backward translation was performed (Prina et al., 2017). In the present study, we tested measurement invariance by comparing HRQL data from respondents in Latin America (Ibero American Spanish) with data collected in the United Kingdom (English). In measurement invariance analyses, group comparisons are made only after matching respondents with the same HRQL estimates based on latent variable models. If group differences are found despite these matched comparisons, this suggests that U.K. and Latin American respondents differ in the way they appraise HRQL when there is no genuine difference in HRQL. As is recommended for ordered categorical data (Rhemtulla, Brosseau-Liard, & Savalei, 2012), all latent variable models were estimated using the diagonally weighted least squares estimator with robust standard errors (denoted WLSMV in Mplus). The detail of the sequence of testing is illustrated below for DEMQOL. We provided the Mplus syntax in the online supplemental material.

### Conceptual Meaning: Configural Model

**Figure 1 fig1:**
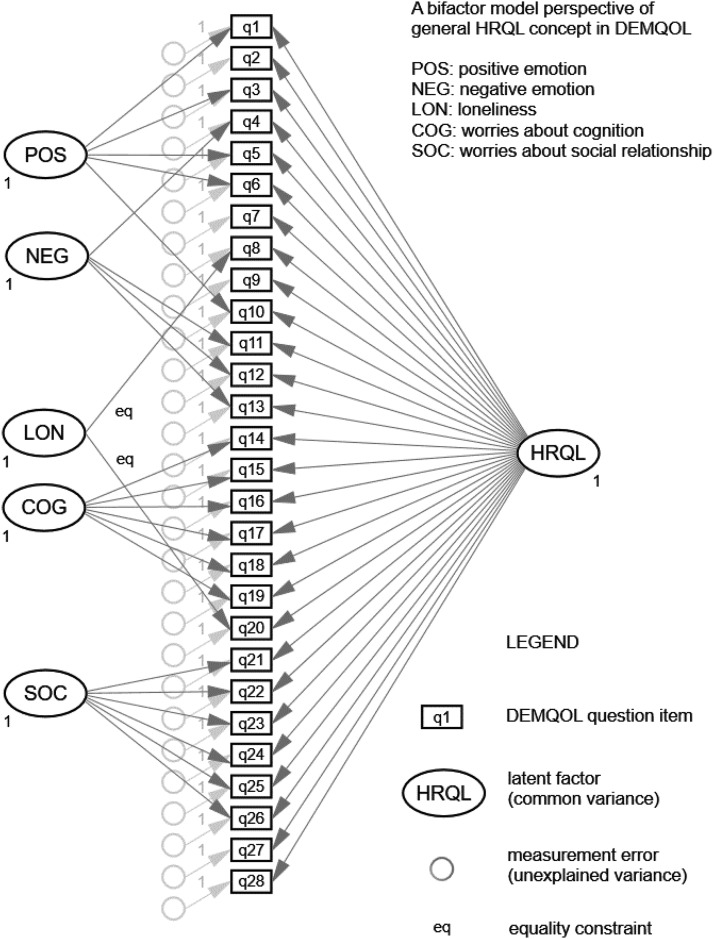
Bifactor model of 28-item DEMQOL (Chua et al., 2016).

The conceptual meaning of HRQL in DEMQOL item responses was first examined using single-group confirmatory factor analytic models (CFA Model 1) for the U.K. and Latin American samples separately. We chose a bifactor model framework (see Figure 1) in which all DEMQOL items load on a general factor as well as orthogonal domain factors that capture additional influence of specific item topics. As general HRQL is commonly treated as the main assessment objective in research and clinical practice (Kifley et al., 2012), the use of a bifactor model helps to retain strategic focus on general HRQL as the target construct for investigating measurement invariance (Chua et al., 2016).[Fig-anchor fig1]

Differences between these configural models provided early indications of how U.K. and Latin American respondents might differ in how they appraise HRQL. Post hoc model modifications were needed such that next stages of statistical comparisons focused on aspects of conceptual meaning that might be identical across language versions of DEMQOL. We modified these configural models based on three considerations: (a) approximate model fit (i.e., small values of root mean square error of approximation [RMSEA; <0.08] and large values of comparative fit index [CFI; >0.90]), (b) precision (i.e., standard errors) of bootstrapped model estimates (Kam & Zhou, 2016; Perera & Ganguly, 2018), and (c) scale reliability (i.e., omega-hierarchical [ω_*h*_] coefficient; Reise, Bonifay, & Haviland, 2013). In calculating reliability for specific domain subscales, ω_*h*_ was modified as ω_*s*_ according to didactic accounts by Reise et al. (2013). However, we used the notation ω_*h*_ for both general and domain specific factor because the same methodological principle applies (Brunner, Nagy, & Wilhelm, 2012) and for ease of presentation. To estimate ω_*h*_, we used standardized factor loadings estimated by WLSMV in line with recent studies across fields of psychological assessment (e.g., Adams et al., 2018; Fergus, Kelley, & Griggs, 2017; Shihata, McEvoy, & Mullan, 2018; Stanton, Forbes, & Zimmerman, 2018).

### Conceptual Meaning: Configural Invariance

To see if DEMQOL items carry the same meaning for U.K. and Latin American participants, direct statistical comparisons between the configural models were made using a multiple-group CFA model. The *factor pattern configuration* refers to the underlying conceptual or cognitive frame of reference used to make item responses (Vandenberg & Lance, 2000). *Configural invariance* (Horn & McArdle, 1992) refers to an invariant pattern of factor loadings, which means that the same DEMQOL items can be grouped under identical domain themes of HRQL for the U.K. and Latin American study samples. This means that respondent groups (U.K. or Latin American) were using the same conceptual frame of reference that reflect equivalent underlying constructs (Vandenberg & Lance, 2000). If the factor loading pattern differs between United Kingdom and Latin America, the concepts that are represented by the common factors do not have the same definition (Oort, 2005). With good approximate model fit according to RMSEA and CFI values, configural invariance (Model 2a) would form the basis for saying that the conceptual meaning of HRQL is the same for both groups. Of note, a HRQL construct must carry the same meaning across groups (configural invariance) before it makes sense to examine if specific aspects are equally sensitive to individual differences in HRQL (metric invariance), and hence after also appraised according to the same internal standards (scalar invariance).

### Sensitivity and Relevance: Metric and Scalar Invariance

To see if DEMQOL item scores were equally sensitive to individual differences in HRQL for the U.K. and Latin American respondents, we compared item factor loadings between the two language versions. Item factor loadings reflect the magnitude of difference in DEMQOL item scores between two individuals given their differences in HRQL. *Metric invariance* (Horn & McArdle, 1992) refers to invariant magnitude of factor loadings which means that DEMQOL items load on the same factors with the same factor loading values giving rise to identical units of measurement in the U.K. and Latin American study sample. This means that DEMQOL item scores would show the same amount of differences for U.K. or Latin American respondents given any two scenarios (e.g., poor vs. average HRQL). If the factor loading value of a DEMQOL item differs between the two study samples, then that item is more (or less) “indicative” of individual differences in HRQL due to different units of measurement between the groups (Oort, 2005). In other words, for one of the groups, the DEMQOL item is more (or less) sensitive to individual differences in HRQL. With good approximate model fit according to RMSEA and CFI values, metric invariance would form the basis for saying that the various aspects are equally sensitive to HRQL for both groups.

To see if DEMQOL items were equally relevant for U.K. and Latin American participants, we compared item thresholds between the two language versions. Item thresholds reflects item difficulty in rating “a lot”/“quite a bit”/“a little”/“not at all” for a DEMQOL item. A “difficult” item is relevant only for individuals with good HRQL. Those with poor HRQL will consistently have low item scores and thus little meaningful differences between these individuals can be observed. Conversely, an “easy” item is relevant only for individuals with poor HRQL. Those with good HRQL will consistently have high item scores and thus little meaningful differences between these individuals can be observed. *Scalar invariance* (Meredith, 1993) refers to invariant item thresholds which means that DEMQOL items load on the same factors with the same factor loading and threshold values in the U.K. and Latin American study sample. This means that internal standards for rating “a lot”/“quite a bit”/“a little”/“not at all” would be calibrated based on the same measurement origins for both groups (Oort, 2005). With good approximate model fit according to RMSEA and CFI values, scalar invariance would form the basis for saying that DEMQOL items are equally relevant for both groups because the same standards of good/poor HRQL apply (i.e., items are equally “difficult” or “easy” for both groups).

Metric and scalar invariance were tested concurrently (e.g., DuPaul et al., 2016; Fergus et al., 2017; Schroeders & Wilhelm, 2011) because item factor loadings and thresholds are mathematically interdependent (Muthén & Asparouhov, 2002; Muthén & Christoffersson, 1981). If both hypotheses were tenable, Model 2b would show good approximate fit according to RMSEA and CFI values. In addition, if Model 2b showed only trivial decline in model fit despite making more assumptions than Model 2a, this would offer stronger support for measurement invariance between versions of DEMQOL. Compared to Model 2a, the decline in exact model fit of Model 2b should not attain statistical significance based on DIFFTEST in Mplus (Asparouhov & Muthén, 2006). The decline in approximate model fit would also be considered trivial if the increases in RMSEA value was less than 0.015 and the decreases in CFI less than 0.010 (Chen, 2007; Cheung & Rensvold, 2002).

### Item Characteristics

We used the combined data from U.K. and Latin American respondents to gain insights on the sensitivity and relevance of DEMQOL items. We estimated these properties with a graded-response model (Model 3), which corresponds mathematically to the CFA configural model we estimated (Kamata & Bauer, 2008). Item response theory (IRT) parameters from Model 3 were plotted to give item information curves to show (a) the level of sensitivity of a DEMQOL item (*y*-axis: discrimination parameters) and (b) how this sensitivity depends on whether people have below-average, average, and above-average HRQL (*x*-axis: difficulty parameters).

## Results

### Conceptual Meaning: Configural Model

All configural models showed acceptable to good fit (see Table 2), but the two samples did not show exactly the same patterns of item response. Comparing the DEMQOL single-group CFA Model 1a between the U.K. and Latin American samples (Supplemental Tables S1 and S2 in the online supplemental material) both had item response patterns that indicated the presence of a general HRQL factor. The U.K. sample had five additional sources of influence: positive emotion (POS), negative emotion (NEG), loneliness (LON), worries about cognition (COG), and worries about social relationship (SOC), but in the Spanish version of DEMQOL, the POS item loadings on the general HRQL factor and the NEG domain loadings were largely not statistically significant. These apparent differences between the United Kingdom and Latin America may not be major because the factor loadings are also relatively weak in the U.K. sample but might have attained statistical significance due to larger sample size.[Table-anchor tbl2]

For DEMQOL-Proxy, the same analyses showed that both groups had item response patterns that indicated the presence of a general HRQL factor (Supplemental Tables S3 and S4 in the online supplemental material). The U.K. sample had six additional sources of influence: POS, NEG, COG, SOC, and worries about finance-related tasks (FIN) and physical appearance (APP), but in the Spanish version POS item loadings on the general HRQL factor were weak and negative despite attaining statistical significance. To ensure that POS item responses were coded in the right direction for the present analyses, we repeated multiple checks and also consulted with the data owners (both for the memory clinic and 10/66 Dementia Research Group). We concluded that the coding was done correctly across the two data sets. Additional reassurance can be found in a body of literature documenting similar influences of positive and negative item wording effects on the measurement of health (Böhnke & Croudace, 2016; Lai, Garcia, Salsman, Rosenbloom, & Cella, 2012; Molina, Rodrigo, Losilla, & Vives, 2014; van Sonderen, Sanderman, & Coyne, 2013) as well as psychological traits (Marsh, 1986; Ray, Frick, Thornton, Steinberg, & Cauffman, 2016; Tomas, Oliver, Galiana, Sancho, & Lila, 2013; Weijters, Baumgartner, & Schillewaert, 2013). Of note, a recent study by an independent group of researchers used a different analytic approach (Rasch modeling) but reach similar conclusions about POS items in DEMQOL and DEMQOL-Proxy (Hendriks, Smith, Chrysanthaki, Cano, & Black, 2017). As POS items are the only reverse-worded items in DEMQOL and DEMQOL-Proxy, these model results are consistent with the presence of wording effects. Even in the U.K. sample, POS items showed the weakest factor loadings on the general HRQL factor (Chua et al., 2016).

To see if POS items were the main difference between configural models, we examined configural models (Model 1b) for the remaining 23 DEMQOL items and 26 DEMQOL-Proxy items, which resulted in good fit for both measures and study samples (see Table 2). This analysis revealed an additional difference between the two language versions. For Latin America respondents, the NEG domain factor in DEMQOL and the SOC domain factor in DEMQOL-Proxy showed signs of “factor collapse” (Chen, West, & Sousa, 2006) as indicated by weak and/or nonstatistically significant loadings on the domain factors (Supplemental Tables S5–S8 in the online supplemental material). This is a statistical indication that responses to these domain items do not share any additional common variance (i.e., common theme) over and above the general theme of HRQL. Consequently, these items have sizable factor loadings only on the general HRQL factor and an additional domain factor is not retained. Even in the U.K. sample, these domain factors also had only a weak impact on item responses as reflected by poor scale reliability (ω_*h*_ = 0.33 for self-report NEG and informant-report SOC). Despite these differences in weaker sources of influence, the dominant impact of general HRQL on item responses was evident across both language versions of DEMQOL (ω_*h*_ = 0.87–0.90) and DEMQOL-Proxy (ω_*h*_ = 0.88–0.89).

We therefore fitted a DEMQOL bifactor model (**Model 1c**) without a NEG domain factor (Supplemental Tables S9 and S10 in the online supplemental material). For DEMQOL-Proxy, we fitted a bifactor model without a SOC domain factor (Supplemental Tables S11 and S12 in the online supplemental material). Based on Brunner et al.’s (2012) substantive interpretations of “factor collapse,” we hypothesized that negative emotion is a core component of general HRQL when appraised by self-report (DEMQOL Model 1c). This is analogous to the hypothesis in cognitive psychology that ‘reasoning ability’ does not convey additional information (i.e., does not exist as an independent domain in a bifactor model) beyond what it conveys about individual differences in general intelligence because performance on this ability test essentially reflects only general intelligence (Gottfredson, 1997; Snow, Kyllonen, & Marshalek, 1984). In other words, responses to NEG items differ between individuals mainly because of their differences in general HRQL. Similarly, “worries about social relationship” is a core component of general HRQL when appraised by informants (DEMQOL-Proxy Model 1c). These models showed adequate to good fit (see Table 2) and were used as the configural model for measurement invariance testing.

### Conceptual Meaning: Configural Invariance

The multiple-group CFA Model 2a directly tested configural invariance between language versions of DEMQOL and DEMQOL-Proxy. The results showed good model fit (see Table 2) when we assumed the same conceptual meaning (i.e., factor loading patterns) for both language versions of DEMQOL and DEMQOL-Proxy (Supplemental Tables S13–S16 in the online supplemental material).

### Sensitivity and Relevance: Metric and Scalar Invariance

When metric and scalar invariance were tested in tandem, Model 2b showed good model fit (see Table 2) for DEMQOL (see Table 3) and DEMQOL-Proxy (see Table 4). These results show it was tenable to assume that the items were equally sensitive to HRQL differences and relevant across both language versions.[Table-anchor tbl3][Table-anchor tbl4]

Compared to Model 2a, the decline in exact model fit of Model 2b according to the DIFFTEST attained statistical significance (see Table 2). However, the decline in approximate model fit was considered trivial for DEMQOL (RMSEA: 0.063 vs. 0.063 and CFI: 0.943 vs. 0.935) and DEMQOL-Proxy (RMSEA: 0.059 vs. 0.059 and CFI: 0.952 vs. 0.943; Chen, 2007; Cheung & Rensvold, 2002). Taken together, these criteria lend support for the tenability of measurement invariance (Model 2b).

### Item Characteristics

With tenable support for measurement invariance in a subset of DEMQOL and DEMQOL-Proxy items, we used the combined U.K. and Latin American data to estimate an IRT graded response model (Model 3). In this model linguistic group was treated as an external covariate predicting differences in latent means between U.K. and Latin American respondents. IRT parameters from Model 3 (Supplemental Tables S17 and S18 in the online supplemental material) were plotted to give item information curves.

**Figure 2 fig2:**
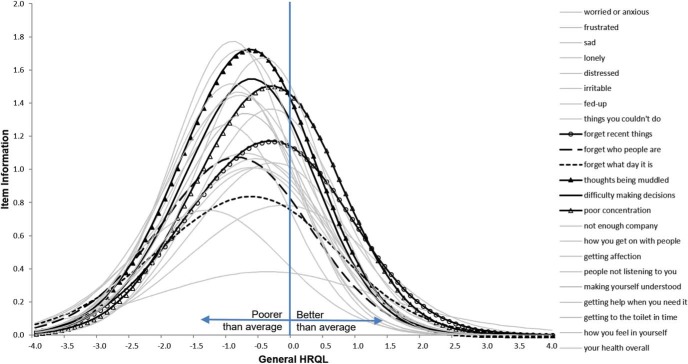
Item information curves of six “worries about cognition” items for self-report HRQL (DEMQOL Model 3). HRQL = health related quality of life.

**Figure 3 fig3:**
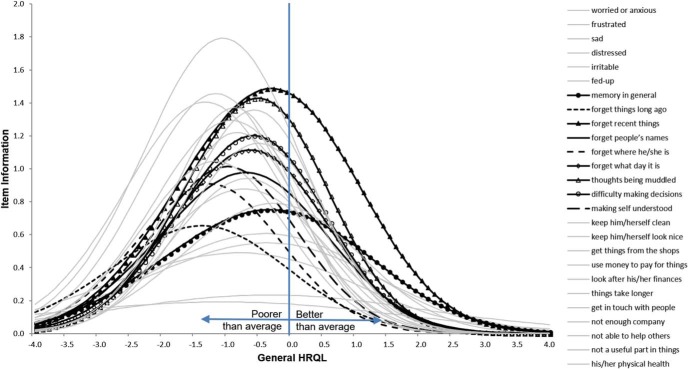
Item information curves of nine “worries about cognition” items for informant-report HRQL (DEMQOL-Proxy Model 3). HRQL = health related quality of life.

Figures 2 and 3 show the item information curves for “worries about cognition” items in DEMQOL and DEMQOL-Proxy items respectively. Across the *x*-axis, latent model estimates of HRQL (general factor; Model 3) are standardized so that sample average is located at the mean of 0 with a standard deviation of 1. The sensitivity (*y*-axis) of most DEMQOL and DEMQOL-Proxy items rises to the highest level at around 1 *SD* below the sample average HRQL (*x*-axis). This means DEMQOL and DEMQOL-Proxy measurements were most sensitive for detecting HRQL differences between people with below average HRQL. [Fig-anchor fig2][Fig-anchor fig3]

Among 23 DEMQOL items, responses about “worries about cognition” (see Figure 2) as well as “negative emotion” (Supplemental Figure S2A in the online supplemental material) were more sensitive for detecting HRQL differences between people with above average HRQL (i.e., 1 *SD* above sample average). Items for “worries about social relationship” (Supplemental Figure S2B in the online supplemental material) were particularly useful for detecting HRQL differences between people with below average HRQL. However, Item 26 (getting to toilet in time) was easy even for respondents with significant HRQL impairment to report “not at all” worried about this matter, so this item was mainly relevant for severe impairment (over 1 *SD* below average). Most other items (Supplemental Figure S2C and S2D in the online supplemental material) show similar levels of sensitivity and standards of difficulty.

Among 26 DEMQOL-Proxy items, informant ratings of “worries about cognition” (see Figure 3) and “worries about social relationship” (Supplemental Figure S3a in the online supplemental material) were more sensitive for detecting HRQL differences between people with above average HRQL. “Negative emotion” items show the least sensitivity across HRQL levels (Figure S3b in online supplement material). Previous research suggested that affective states are less easily observed by informants (Novella et al., 2001). For worries about “finance-related tasks” and “physical appearance” (Supplemental Figure S3C–S3D in the online supplemental material) most informants would rate “not at all” on these items, so they are mainly relevant for assessing severe impairment (over 1 *SD* below average).

## Discussion

The main finding of this study is that the data offer the first empirical support for the use of a dementia-specific measure of HRQL cross-culturally, in this case the use of the DEMQOL system in the United Kingdom and Latin America. This is supported by our psychometric evaluation which found strong measurement invariance for the general HRQL factor, the dominant influence on item responses from self- and informant-reports. We can therefore conclude that DEMQOL and DEMQOL-Proxy carry the same meaning, sensitivity, and relevance for respondents in the United Kingdom and Latin America. However, differences in domain factors suggest benefits in making statistical adjustments for weaker influences on item responses. Also, these cross-cultural comparisons provide new insights on HRQL measurement in dementia, showing that “negative emotion” is a core component in self-reports and “worries about social relationship” in informant-reports.

Lawton (1994) postulated that the absence of HRQL impairment is not the same as good HRQL and that HRQL is a construct “concerned primarily with decrements from the average.” Our construct validation study supports Lawton’s position. We found that “positive emotion” was not a major component of general HRQL in the Latin American sample, a similar pattern to that found in U.K. samples (Chua et al., 2016). If the absence of “HRQL impairment” is not the same as “good HRQL,” then HRQL impairment might be considered a unipolar construct (Reise & Waller, 2009) in which the presence of impairment shows meaningful individual differences, but the absence of impairment gives little insight about what constitutes “good” HRQL. This is in line with the finding in the general HRQL measurement literature that negative and positive components of well-being may be different or partly independent aspects of people’s experience (Böhnke & Croudace, 2016).

A further explanation for the factor loadings may be that there are wording effects for the “positive emotion” because they are reverse-worded items in DEMQOL and DEMQOL-Proxy. In the absence of HRQL impairment (i.e., “good” HRQL), one may find it easy to respond “not at all” when asked if he or she has “worries” but not as easy to respond “very much” when asked if he or she is “feeling cheerful”. This is consistent with findings from a U.K. population-based study which showed an asymmetry between strong adverse reactions to deteriorations in health, alongside weak increases in well-being after health improvements (Binder & Coad, 2013). Such wording effects may have unequal strengths in different languages. Consideration should be given to these issues in the development and translation of instruments for cross-cultural use.

Further discussion, informed by evidence, is needed before POS items can be recommended for exclusion from the questionnaire. Our findings do not mean that positive states are not important for general HRQL. Lawton (1994) proposed that, for people with dementia, indicators of positive states may be found in both positive affective states and positive behaviors, such as behaviors that exemplify social engagement. As such DEMQOL and DEMQOL-Proxy consider positive states as part of general HRQL by tapping on items that focus on “worries about social relationship.” This focus contributes to the clinical relevance of HRQL assessment as social functioning is “a treatment goal that seems appropriate for an illness whose manifestations in general appear to represent estrangement from the external world” (Lawton, 1994). Such a focus is also consistent with a large body of literature demonstrating that social functioning plays a pivotal role in the illness experience (Frick, Irving, & Rehm, 2012; Lou, Chi, Kwan, & Leung, 2013; MacRae, 2011; T. F. Hughes, Flatt, Fu, Chang, & Ganguli, 2013) as well as healthy aging in general (Coyle & Dugan, 2012; Huxhold, Fiori, & Windsor, 2013; Ichida et al., 2013; Rook, Luong, Sorkin, Newsom, & Krause, 2012).

### Study Limitations

This study has three important limitations. First, although the high overall sample size for the Latin American countries was appropriate for invariance analyses (*n* = 417 for DEMQOL and *n* = 495 for DEMQOL-Proxy), the numbers in individual country samples were relatively small (between *n* = 56 for DEMQOL in Venezuela and *n* = 125 for DEMQOL-Proxy in the Dominican Republic). We therefore carried out pooled analyses for the Latin American sample on the basis that the same translation was used, however this means we cannot comment on between-country differences. Second, the samples were recruited using different processes in the U.K. and Latin American sites. The former was from a memory assessment service (Banerjee et al., 2007) and the latter from a program of population research (Prince et al., 2007). However, all had well-characterized diagnoses of dementia and statistically matched comparisons were used. Third, although our sequence of models was based on established strategies to test for measurement invariance (Vandenberg & Lance, 2000), modeling decisions were data-driven and need replication in independent samples to guard against sample-based overfitting (Borsboom, 2006). Of note, future studies should consider a priori use of bifactor (S-1) models (Eid, Geiser, Koch, & Heene, 2017) to help clarify if negative emotion and/or worries about social functioning constitute the core meaning of HRQL in dementia (Heinrich, Zagorscak, Eid, & Knaevelsrud, 2018). Nevertheless, the study also has strengths. We assembled a unique dataset which allowed for the novel investigation of a key concern in global dementia research (Dichter et al., 2016). Our findings align with empirical literature that shows that even for well-developed measures with translation processes that follow best-practice guidelines, international comparability is not a straightforward issue (Romppel et al., 2017; Stevanovic et al., 2017; Yao et al., 2018) but that there is room for optimism that HRQL measures can be used cross-culturally in dementia.

### Conclusions

Treatment and policy interventions that improve the lives of people with dementia carry both societal and fiscal impact. The stakes are particularly high in world regions like Latin America where the global burden of dementia is high and growing quickly (Prince, Wimo, et al., 2015). To develop global strategies, HRQL assessment is therefore as needed in low- and middle-income countries as it is in high-income countries. The lack of research resources in low- and middle-income regions like Latin America (Barreto et al., 2012) is a key challenge to developing an evidence base on interventions in dementia that is relevant to the countries in which they may be deployed. This study presents the first in-depth study of the cross-cultural assessment of HRQL and shows that, with care, using translated instruments can generate meaningful insights. This is an important step on the path to developing a firm empirical basis for the benefits of dementia interventions in low- and middle-income countries as well as future global trials.

## Supplementary Material

10.1037/pas0000743.supp

## Figures and Tables

**Table 1 tbl1:** Demographic and Clinical Characteristics of Respondents

Variable	DEMQOL		DEMQOL-Proxy	
	United Kingdom (*n* = 868)	Latin America^a^ (*n* = 417)	United Kingdom (*n* = 909)	Latin America^b^ (*n* = 495)
Age (*SD*)	78.6 (8.5)	79.7 (7.6)	78.9 (8.4)	79.7 (7.7)
Gender				
Male	313	126	340	138
Female	555	291	569	357
Severity				
Mild	MMSE > 20	CDR < 2	MMSE > 20	CDR < 2
	517	353	509	392
Moderate	MMSE 15–20	CDR = 2	MMSE 15–20	CDR = 2
	255	50	268	77
Severe	MMSE < 15	CDR = 3	MMSE < 15	CDR = 3
	96	14	132	26
*Note*. MMSE = Mini-Mental State Examination (Folstein, Folstein, & McHugh, 1975); CDR = Clinical Dementia Rating Scale (C. P. Hughes, Berg, Danziger, Coben, & Martin, 1982). Age differences between United Kingdom and Latin America study samples have small effect sizes for DEMQOL (Hedges’ *g* = .13) and DEMQOL-Proxy (Hedges’ *g* = .10). Differences in proportion of male respondents between UK and Latin America study samples have small effect sizes for DEMQOL (Phi = .06) and DEMQOL-Proxy (Phi = .10). Differences in illness severity prevalence between United Kingdom and Latin America study samples have medium effect sizes for DEMQOL (Cramer’s V = .25) and DEMQOL-Proxy (Cramer’s V = .23).				
^a^ Venezuela (*n* = 53), Peru (*n* = 70), Cuba (*n* = 90), Mexico (*n* = 87), and Dominican Republic (*n* = 117). ^b^ Venezuela (*n* = 65), Peru (*n* = 92), Cuba (*n* = 110), Mexico (*n* = 103), and Dominican Republic (*n* = 125).				

**Table 2 tbl2:** Model Fit Indices

Variable	DEMQOL (English)			DEMQOL-Proxy (English)			DEMQOL (Spanish)			DEMQOL-Proxy (Spanish)		
	Model χ^2^	RMSEA [90% CI]	CFI	Model χ^2^	RMSEA [90% CI]	CFI	Model χ^2^	RMSEA [90% CI]	CFI	Model χ^2^	RMSEA [90% CI]	CFI
Single-group CFA												
Model 1a (*df* = 328)	1,420.582	.062 [.059, .065]	.918				738.266	.055 [.050, .060]	.958			
Model 1b (*df* = 213)	793.336	.056 [.052, .060]	.946				531.840	.060 [.054, .066]	.964			
Model 1c (*df* = 217)	664.715	.064 [.060, .068]	.928				568.640	.062 [.056, .069]	.961			
Multiple-group CFA^a^												
Model 2a (*df* = 434)	1,553.474	.063 [.060, .067]	.943									
Model 2b (*df* = 508)	1,788.347	.063 [.060, .066]	.935									
Single-group CFA												
Model 1a (*df* = 406)				1,647.018	.058 [.055, .061]	.932				1,133.244	.060 [0 .056, .064]	.950
Model 1b (*df* = 277)				715.923	.042 [.038, .046]	.972				912.963	.068 [0 .063, .073]	.951
Model 1c (*df* = 281)				1,000.566	.053 [.050, .057]	.954				944.623	.069 [0 .064, .074]	.948
Multiple-group CFA^b^												
Model 2a (*df* = 562)				1,930.082	.059 [.056, .062]	.952						
Model 2b (*df* = 648)				2,254.917	.059 [.057, .062]	.943						
*Note*. RMSEA = root mean square error of approximation; CFA = confirmatory factor analytic; CFI = comparative fit index. Model 1a: Configural model for 28-item DEMQOL and 31-item DEMQOL-Proxy. Model 1b: Configural model (without POS items) for 23-item DEMQOL and 26-item DEMQOL-Proxy. Model 1c: Configural model for 23-item DEMQOL (without NEG domain) and 26-item DEMQOL-Proxy (without social relationship [SOC] domain). Model 2a: Configural invariance. Model 2b: Configural, metric, scalar invariance.												
^a^ Model 2a vs 2b: DIFFTEST χ^2^ = 325.029 (Δ*df* = 74), *p* < .0001 for DEMQOL. ^b^ Model 2a vs. 2b: DIFFTEST χ^2^ = 405.844 (Δ*df* = 86), *p* < .0001 for DEMQOL-Proxy.												

**Table 3 tbl3:** Multiple-Group CFA (Model 2b) for 23 DEMQOL Items (Unstandardized Factor Loadings With Bootstrapped Standard Errors): Scalar Invariance Estimates

Item	DEMQOL (*n* = 1,284), EL (*n* = 867), ES (*n* = 417)	h^2^	GEN	*SE*	COG	*SE*	LON	*SE*	SOC	*SE*
2	Worried or anxious	.38	.97	*.05*						
4	Frustrated	.41	.89	*.04*						
7	Sad	.39	.95	*.04*						
8	Lonely	.59	.73	*.05*			1.00	^a^		
9	Distressed	.37	1.12	*.05*						
11	Irritable	.42	.90	*.04*						
12	Fed-up	.49	1.00	^a^						
13	Things to do but couldn’t	.46	.70	*.05*						
14	Forget recent things	.69	.85	*.05*	1.07	*.11*				
15	Forgetting who people are	.78	.80	*.05*	.96	*.09*				
16	Forgetting what day it is	.70	.72	*.05*	1.00	^a^				
17	Your thoughts being muddled	.79	.95	*.05*	1.03	*.09*				
18	Difficulty making decisions	.71	1.01	*.05*	.79	*.10*				
19	Poor concentration	.74	.95	*.05*	.82	*.11*				
20	Not having enough company	.77	.74	*.05*			1.00	^a^		
21	Get on with people close	.70	.90	*.07*					.70	*.13*
22	Getting affection that you want	.68	.89	*.07*					.95	*.16*
23	People not listening to you	.87	.86	*.06*					1.00	^a^
24	Making yourself understood	.82	.85	*.06*					.77	*.07*
25	Getting help when you need it	.73	.99	*.06*					.77	*.09*
26	Getting to the toilet in time	.50	.72	*.07*					.61	*.11*
27	How you feel in yourself	.59	1.08	*.05*						
28	Your health overall	.44	.91	*.05*						
	Factor variance (EL)		.48		.23		.53		.32	
	Factor variance (ES)		1.52		.88		.43		.48	
	Factor means (ES)		.35		.16^ns^		−.48		−.51	
*Note*. GEN = general HRQL; COG = worries about cognition; LON = loneliness; SOC = worries about social relationship. Model fit from non-bootstrapped results: χ^2^ = 1,788.347 (*df* = 508), English (EL) χ^2^ = 983.708, Spanish (ES) χ^2^ = 804.639. Root mean square error of approximation (RMSEA) = .063 (90% confidence interval [CI] = .060, .066), comparative fit index (CFI) = .935. DIFFTEST χ^2^ = 325.029 (Δ*df* = 74), *p* < .0001. h^2^ = communalities.										
^a^ Unstandardized factor loading fixed at value of 1. Numbers in italics are values of Standard Error (*SE*).										

**Table 4 tbl4:** Multiple-Group CFA (Model 2b) for 26 DEMQOL-Proxy Items (Unstandardised Factor Loadings With Bootstrapped Standard Errors): Scalar Invariance Estimates

Item	DEMQOL (*n* = 1404), DEMQOL-Proxy EL (*n* = 909), DEMQOL-Proxy ES (*n* = 495)	h^2^	GEN	*SE*	NEG	*SE*	APP	*SE*	FIN	*SE*	COG	*SE*
2	Worried or anxious	.57	.73	*.05*	1.00	^a^						
3	Frustrated	.56	.64	*.05*	1.21	*.11*						
5	Sad	.52	.71	*.06*	1.20	*.10*						
7	Distressed	.43	.84	*.06*	1.01	*.08*						
9	Irritable	.26	.47	*.06*	1.07	*.12*						
10	Fed-up	.27	.80	*.05*	1.06	*.10*						
12	Memory in general	.60	.76	*.06*							1.00	^a^
13	Forget long ago things	.71	.68	*.06*							.79	*.08*
14	Forget recent things	.78	.88	*.06*							1.21	*.07*
15	Forget people’s names	.80	.77	*.05*							.81	*.08*
16	Forget where he/she is	.73	.77	*.06*							.60	*.08*
17	Forget what day it is	.68	.87	*.05*							.75	*.08*
18	Thoughts muddled	.71	.98	*.05*							.81	*.10*
19	Difficulty deciding	.68	.94	*.05*							.76	*.10*
20	Making self understood	.58	.87	*.05*							.57	*.10*
21	Keeping clean	.89	.76	*.07*			1.00	^a^				
22	Keeping looking nice	.94	.78	*.06*			1.00	^a^				
23	Get things from shops	.72	.88	*.05*					1.00	^a^		
24	Using money to pay	.88	.88	*.06*					1.31	*.12*		
25	Looking after finances	.85	.83	*.06*					1.31	*.12*		
26	Things take longer	.49	.95	*.05*								
27	Get in touch with people	.57	1.00	^a^								
28	Not enough company	.40	.88	*.05*								
29	Not being able to help	.69	.91	*.04*								
30	Not playing a useful part	.68	.92	*.05*								
31	His/her physical health	.53	.72	*.05*								
	Factor variance (EL)		.52		.25		.51		.21		.28	
	Factor variance (ES)		1.80		1.15		1.66		1.04		2.43	
	Factor means (ES)		.12^*ns*^		.84		−.87		.20^ns^		1.13	
*Note*. GEN = general HRQL; NEG = negative emotion; APP = worries about physical appearance; FIN = worries about finance-related tasks; COG = worries about cognition. Model fit from non-bootstrapped results: χ^2^ = 2,254.917 (*df* = 648), English (EL) χ^2^ = 934.728, Spanish (ES) χ^2^ = 1,320.188. Root mean square error of approximation (RMSEA) = .059 (90% confidence interval [CI] = .057, .062), comparative fit index (CFI) = .943. DIFFTEST χ^2^ = 405.844 (Δ*df* = 86), *p* < .0001. h^2^ = communalities.												
^a^ Unstandardized factor loading fixed at value of 1. Numbers in italics are values of Standard Error (*SE*).
