# Supplementary material for: Health-Related Quality-of-Life Assessment in Dementia: Evidence of Cross-Cultural Validity in Latin America
Source: Psychol Assess. 2019 Jul 8;31(10):1264–77. doi: 10.1037/pas0000743 (PMC6818683; doi:10.1037/pas0000743)
Supplement: Supplementary file 1 [file PAS-2018-2584_Suppl.pdf]

Chua, Böhnke, Prince & Banerjee. Health-related quality-of-life assessment in dementia: evidence of cross-cultural validity in Latin America. Psychological Assessment.

ONLINE SUPPLEMENT

**Page 1**	**Table of Contents**
Page 2	Reporting of Model 1a results
Page 3	Figure 1
Page 4	Mplus syntax for 28-item DEMQOL Model 1a
Page 5	Table S1 Bifactor CFA (Model 1a) for English version of 28-item DEMQOL
Page 6	Table S2 Bifactor CFA (Model 1a) for Spanish version of 28-item DEMQOL
Page 7	Figure S1 Bifactor model of 31-item DEMQOL-Proxy
Page 8	Mplus syntax for 31-item DEMQOL-Proxy Model 1a
Page 9	Table S3 Bifactor CFA (Model 1a) for English version of 31-item DEMQOL-Proxy
Page 10	Table S4 Bifactor CFA (Model 1a) for Spanish version of 31-item DEMQOL-Proxy
Page 11	Reporting of Model 1b results
Page 12	Mplus syntax for 23 items in DEMQOL Model 1b
Page 13	Table S5 Bifactor CFA (Model 1b) for English version of 23 DEMQOL items
Page 14	Table S6 Bifactor CFA (Model 1b) for Spanish version of 23 DEMQOL items
Page 15	Mplus syntax for 26 items in DEMQOL-Proxy Model 1b
Page 16	Table S7 Bifactor CFA (Model 1b) for English version of 26 DEMQOL-Proxy items
Page 17	Table S8 Bifactor CFA (Model 1b) for Spanish version of 26 DEMQOL-Proxy items
Page 18	Reporting of Model 1c results
Page 19	Mplus syntax for 23 items in DEMQOL Model 1c
Page 20	Table S9 Bifactor CFA (Model 1c) for English version of 23 DEMQOL items
Page 21	Table S10 Bifactor CFA (Model 1c) for Spanish version of 23 DEMQOL items
Page 22	Mplus syntax for 26 items in DEMQOL-Proxy Model 1c
Page 23	Table S11 Bifactor CFA (Model 1c) for English version of 26 DEMQOL-Proxy items
Page 24	Table S12 Bifactor CFA (Model 1c) for Spanish version of 26 DEMQOL-Proxy items
Page 25	Reporting of Model 2a results
Page 26	Mplus syntax for 23 items in DEMQOL Model 2a
Page 27	Table S13 Multiple-group CFA (Model 2a) for 23 DEMQOL items
Page 28	Table S14 Multiple-group CFA (Model 2a) for 23 DEMQOL items
Page 29	Mplus syntax for 26 items in DEMQOL-Proxy Model 2a
Page 30	Table S15 Multiple-group CFA (Model 2a) for 26 DEMQOL-Proxy items
Page 31	Table S16 Multiple-group CFA (Model 2a) for 26 DEMQOL-Proxy items
Page 32	Mplus syntax for 23 items in DEMQOL Model 2b
Page 33	Mplus syntax for 26 items in DEMQOL-Proxy Model 2b
Page 34	Mplus syntax for 23 items in DEMQOL Model 3 (MIMIC)
Page 35	Table S17 Bifactor CFA with language groups as external covariate (Model 3) in combined sample (n=1284) data for 23 DEMQOL items
Page 36	Mplus syntax for 26 items in DEMQOL-Proxy Model 3 (MIMIC)
Page 37	Table S18 Bifactor CFA with language groups as external covariate (Model 3) in combined sample (n=1404) data for 26 DEMQOL-Proxy items
Page 38	Figure 2 Item information curves of 6 “worry about cognition” items for self-report HRQL
Page 39	Figure S2a Item information curves of 7 “negative emotion” items for self-report HRQL
Page 40	Figure S2b Item information curves of 6 “worry about social relationship” items for self-report HRQL
Page 41	Figure S2c Item information curves of 2 “loneliness” items for self-report HRQL
Page 42	Figure S2d Item information curves of 2 items that loaded only on general HRQL for self-report HRQL
Page 43	Figure 3 Item information curves of 9 “worry about cognition” items for informant-report HRQL
Page 44	Figure S3a Item information curves of 4 “worry about social relationship” and 2 general HRQL items for informant-report HRQL
Page 45	Figure S3b Item information curves of 6 “negative emotion” items for informant-report HRQL
Page 46	Figure S3c Item information curves of 3 “worry about finance-related tasks” items for informant-report HRQL
Page 47	Figure S3d Item information curves of 2 “worry about physical appearance” items for informant-report HRQL

The single-group bifactor CFA models (**Model 1a**) for DEMQOL (Table S1) and DEMQOL-Proxy (Table S3) in the UK study sample (Chua *et al.*, 2016) showed acceptable to good model fit in the Latin America study sample (Table S2 and S4). However **Model 1a's** bootstrapped estimates in the latter did not support configural invariance. For the Spanish version of DEMQOL (Table S2), "*positive emotion*" (POS) item loadings on the general HRQL factor were largely not statistically significant. For the Spanish version of DEMQOL-Proxy (Table S4), POS item loadings on the general HRQL factor were statistically significant but in a weak negative direction. As noted by Chua *et al.* (2016) in the UK study sample (Table S1 and S3), POS items are the only reverse-worded items in DEMQOL and DEMQOL-Proxy and the model results for Latin America study sample may be consistent with the presence of 'wording effects'.

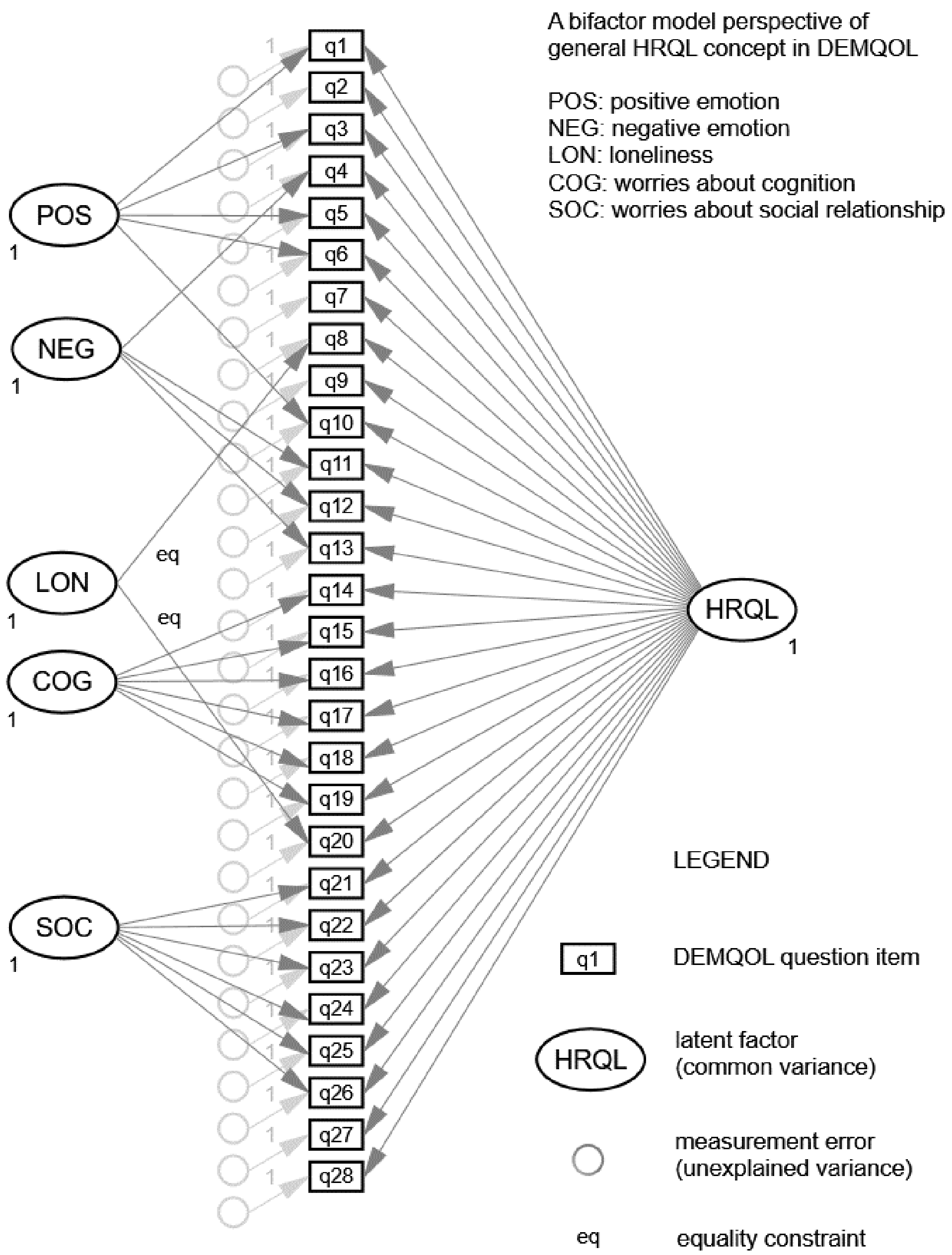

Figure 1 Bifactor model of 28-item DEMQOL

(presented in main paper, reproduced here for ease of reference)

Mplus syntax for 28-item **DEMQOL** Model 1a (Table S1 and S2)

```
Title:
single-group bifactor CFA
for 28-item DEMQOL
UK (EL) vs Latin America (ES)

Data: File = p2croy1066.dat ;

Variable:
Names = lang f0 sev
a1 a2 a3 a4 a5 a6 a7 a8 a9 a10
a11 a12 a13 a14 a15 a16 a17 a18 a19 a20
a21 a22 a23 a24 a25 a26 a27 a28
b1 b2 b3 b4 b5 b6 b7 b8 b9 b10
b11 b12 b13 b14 b15 b16 b17 b18 b19 b20
b21 b22 b23 b24 b25 b26 b27 b28 b29 b30
b31 ;

Missing = all (-1234) ;
Usev = a1 - a28  ;

Categorical = all  ;

!! single-group CFA
Useobs = lang eq 0  ;

!! multiple-group CFA
!Grouping = lang (0=EL 1=ES) ;

Analysis:
BOOTSTRAP = 5000  ;
```

```
Model:
gen by
a1* a2 a3 a4 a5 a6 a7 a8 a9 a10
a11 a12 a13 a14 a15 a16 a17 a18 a19 a20
a21 a22 a23 a24 a25 a26 a27 a28 ;

pos by a1* a3 a5 a6 a10 ;
neg by a4* a11 a12 a13 ;
cog by a14* a15 a16 a17 a18 a19 ;
lon by a8* a20 (eq1) ;
soc by a21* a22 a23 a24 a25 a26 ;

!! fix factor variance
gen@1 pos@1 neg@1 cog@1 lon@1 soc@1 ;

!! orthogonality
gen with pos@0 neg@0 cog@0 lon@0 soc@0 ;
pos with neg@0 cog@0 lon@0 soc@0 ;
neg with cog@0 lon@0 soc@0 ;
cog with lon@0 soc@0 ;
lon with soc@0 ;

Output:
!sampstat residual modindices;
CINTERVAL (BCBOOTSTRAP);
```

Table S1 Bifactor CFA (Model 1a) for **English** version of 28-item **DEMQOL** (standardised factor loadings with bootstrapped standard errors)

Qn	DEMQOL (n = 868)	h^2	**GEN**	*SE*	**POS**	*SE*	**NEG**	*SE*	**COG**	*SE*	**LON**	*SE*	**SOC**	*SE*
1	cheerful	.48	.47	*.04*	.51	*.04*								
2	worried or anxious	.48	.70	*.03*										
3	that you are enjoying life	.52	.38	*.04*	.61	*.03*								
4	frustrated	.91	.57	*.04*			.77	*.18*						
5	confident	.44	.40	*.04*	.53	*.04*								
6	full of energy	.66	.31	*.04*	.75	*.03*								
7	sad	.48	.70	*.03*										
8	lonely	.80	.54	*.04*							.71	*.03*		
9	distressed	.63	.79	*.03*										
10	lively	.66	.24	*.04*	.77	*.03*								
11	irritable	.43	.59	*.04*			.29	*.07*						
12	fed-up	.58	.69	*.03*			.32	*.07*						
13	things to do but couldn't	.30	.47	*.04*			.28	*.06*						
14	forget recent things	.60	.58	*.03*					.51	*.04*				
15	forgetting who people are	.49	.52	*.04*					.47	*.05*				
16	forgetting what day it is	.47	.48	*.04*					.49	*.05*				
17	your thoughts being muddled	.74	.62	*.03*					.60	*.04*				
18	difficulty making decisions	.61	.72	*.03*					.32	*.05*				
19	poor concentration	.61	.64	*.03*					.45	*.04*				
20	not having enough company	.79	.53	*.04*							.71	*.03*		
21	get on with people close	.61	.58	*.04*									.53	*.06*
22	getting affection that you want	.76	.57	*.05*									.66	*.06*
23	people not listening to you	.64	.60	*.04*									.53	*.06*
24	making yourself understood	.48	.60	*.04*									.35	*.07*
25	getting help when you need it	.61	.67	*.04*									.39	*.06*
26	getting to the toilet in time	.36	.48	*.05*									.36	*.07*
27	how you feel in yourself	.58	.76	*.02*										
28	your health overall	.40	.63	*.03*										
		ω_h	.85		.65		.28		.35		.57		.35	

Model fit from non-bootstrapped results:
χ^2 = 1420.583 (df = 328), RMSEA = **.062** (90% CI = .059 - .065), CFI = **.918**
h^2: communalities
ω_h: Omega hierarchical coefficient

Table S2 Bifactor CFA (Model 1a) for **Spanish** version of 28-item **DEMQOL** (standardised factor loadings with bootstrapped standard errors)

Qn	DEMQOL (n = 417)	h^2	**GEN**	*SE*	**POS**	*SE*	**NEG**	*SE*	**COG**	*SE*	**LON**	*SE*	**SOC**	*SE*
1	cheerful	.47	.19	*.06*	.66	*.05*								
2	worried or anxious	.38	.62	*.05*										
3	that you are enjoying life	.51	.12^{ns}	*.07*	.71	*.04*								
4	frustrated	.43	.63	*.05*			.18^{ns}	*.10*						
5	confident	.47	.00^{ns}	*.07*	.69	*.04*								
6	full of energy	.59	.03^{ns}	*.07*	.77	*.04*								
7	sad	.41	.64	*.04*										
8	lonely	.59	.60	*.05*							.48	*.06*		
9	distressed	.38	.61	*.05*										
10	lively	.64	.00^{ns}	*.07*	.80	*.03*								
11	irritable	.54	.61	*.05*			.41^{ns}	*.24*						
12	fed-up	.76	.66	*.05*			.57^{ns}	*.34*						
13	things to do but couldn't	.49	.66	*.04*			.22	*.09*						
14	forget recent things	.69	.60	*.05*					.58	*.06*				
15	forgetting who people are	.78	.66	*.05*					.59	*.06*				
16	forgetting what day it is	.70	.58	*.05*					.60	*.05*				
17	your thoughts being muddled	.78	.72	*.04*					.51	*.05*				
18	difficulty making decisions	.70	.67	*.05*					.50	*.06*				
19	poor concentration	.74	.74	*.04*					.44	*.06*				
20	not having enough company	.77	.74	*.04*							.48	*.06*		
21	get on with people close	.70	.82	*.04*									.16^{ns}	*.09*
22	getting affection that you want	.68	.77	*.04*									.28	*.08*
23	people not listening to you	.86	.75	*.04*									.55	*.07*
24	making yourself understood	.82	.71	*.04*									.56	*.07*
25	getting help when you need it	.73	.79	*.04*									.34	*.07*
26	getting to the toilet in time	.50	.64	*.05*									.31	*.09*
27	how you feel in yourself	.59	.77	*.04*										
28	your health overall	.45	.67	*.04*										
		ω_h	.86		.84		.19		.37		.27		.18	

Model fit from non-bootstrapped results:
χ^2 = 738.266 (df = 328), RMSEA = **.055** (90% CI = .050 - .060), CFI = **.958**
h^2: communalities
ns: not statistically significant
ω_h: Omega hierarchical coefficient

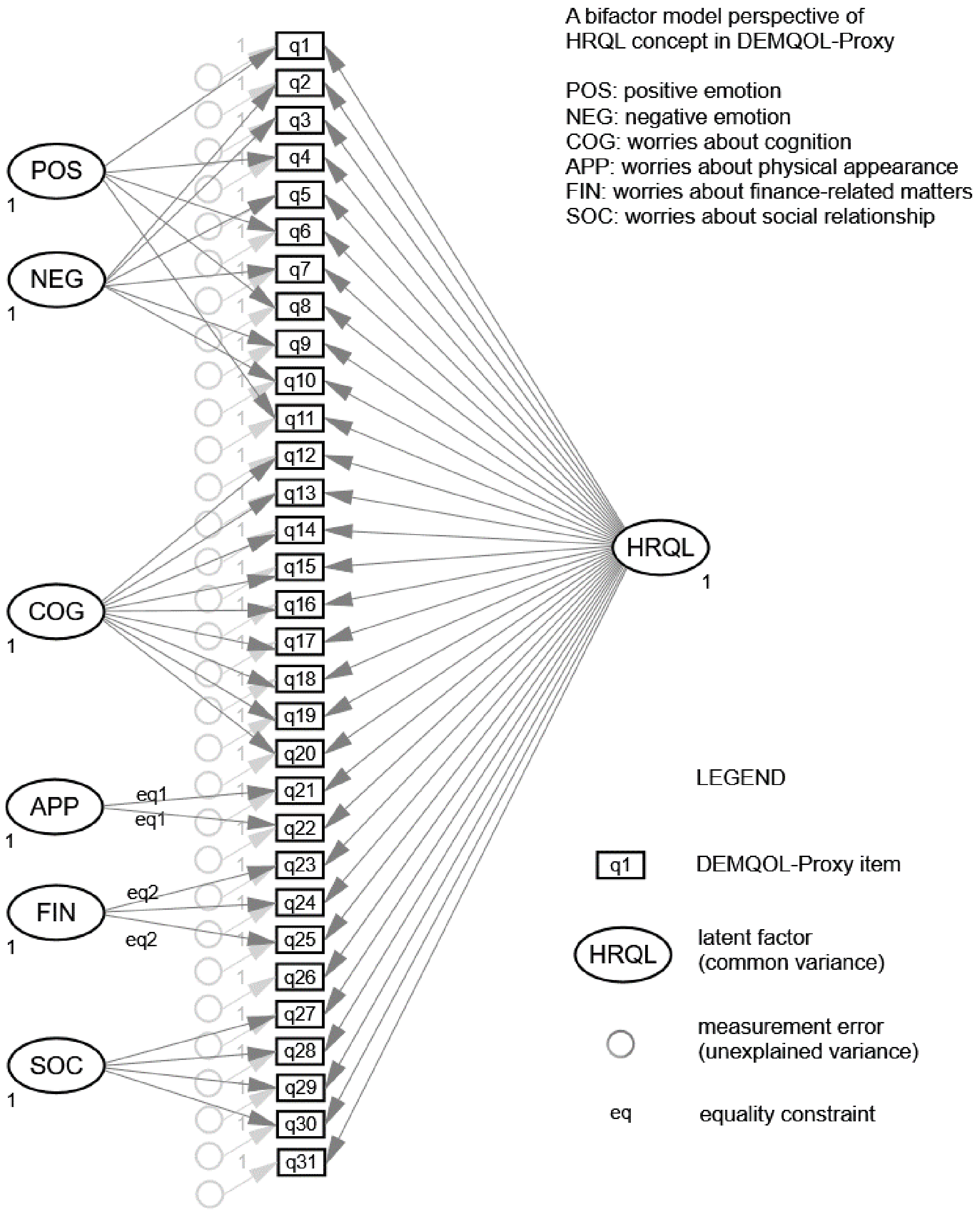

Figure S1 Bifactor model of 31-item DEMQOL-Proxy

Mplus syntax for 31-item **DEMQOL-Proxy** Model 1a (Table S3 and S4)

Title:<br>single-group bifactor CFA<br>for 31-item DEMQOL-Proxy<br>UK (EL) vs Latin America (ES)<br><br>Data: File = p2croy1066.dat ;<br><br>Variable:<br>Names = lang f0 sev<br>a1 a2 a3 a4 a5 a6 a7 a8 a9 a10<br>a11 a12 a13 a14 a15 a16 a17 a18 a19 a20<br>a21 a22 a23 a24 a25 a26 a27 a28<br>b1 b2 b3 b4 b5 b6 b7 b8 b9 b10<br>b11 b12 b13 b14 b15 b16 b17 b18 b19 b20<br>b21 b22 b23 b24 b25 b26 b27 b28 b29 b30<br>b31 ;<br><br>Missing = all (-1234) ;<br>Usev = b1 - b31 ;<br><br>Categorical = all ;<br><br>!! single-group CFA<br>Useobs = lang eq 0 ;<br><br>!! multiple-group CFA<br>!Grouping = lang (0=EL 1=ES) ;<br><br>Analysis:<br>BOOTSTRAP = 5000 ;	Model:<br>gen by<br>b1* b2 b3 b4 b5 b6 b7 b8 b9 b10<br>b11 b12 b13 b14 b15 b16 b17 b18 b19 b20<br>b21 b22 b23 b24 b25 b26 b27 b28 b29 b30<br>b31 ;<br><br>pos by b1* b4 b6 b8 b11 ;<br>neg by b2* b3 b5 b7 b9 b10 ;<br>app by b21* b22 (eq11) ;<br>!fin by b23* b24 b25 ;<br>fin by b23* ;<br>fin by b24* b25 (eq12) ;<br>soc by b27* b28 b29 b30 ;<br>cog by b12* b13 b14 b15 b16 b17 b18 b19 b20 ;<br><br>!! fix factor variance<br>gen@1 pos@1 neg@1 app@1 fin@1 soc@1 cog@1 ;<br><br>!! orthogonality<br>gen with pos@0 neg@0 app@0 fin@0 soc@0 cog@0 ;<br>pos with neg@0 app@0 fin@0 soc@0 cog@0 ;<br>neg with app@0 fin@0 soc@0 cog@0 ;<br>app with fin@0 soc@0 cog@0 ;<br>fin with soc@0 cog@0 ;<br>soc with cog@0 ;<br><br>Output:<br>!sampstat residual modindices;<br>CINTERVAL (BCBOOTSTRAP);

Table S3 Bifactor CFA (Model 1a) for **English** version of 31-item **DEMQOL-Proxy** (standardised factor loadings with bootstrapped standard errors)

Qn	DEMQOL-Proxy (n = 909)	h^2	**GEN**	*SE*	**POS**	*SE*	**NEG**	*SE*	**APP**	*SE*	**FIN**	*SE*	**SOC**	*SE*	**COG**	*SE*
1	cheerful	.45	.39	*.04*	.55	*.03*										
2	worried or anxious	.51	.57	*.03*			.43	*.05*								
3	frustrated	.51	.56	*.03*			.44	*.04*								
4	full of energy	.70	.19	*.04*	.82	*.02*										
5	sad	.60	.61	*.04*			.48	*.05*								
6	content	.45	.48	*.03*	.47	*.04*										
7	distressed	.65	.64	*.04*			.49	*.05*								
8	lively	.78	.21	*.04*	.86	*.03*										
9	irritable	.41	.40	*.04*			.50	*.06*								
10	fed-up	.63	.66	*.03*			.44	*.04*								
11	things to look forward	.35	.26	*.04*	.53	*.04*										
12	memory in general	.62	.47	*.04*											.63	*.03*
13	forget long ago things	.37	.46	*.05*											.40	*.05*
14	forget recent things	.86	.57	*.03*											.73	*.03*
15	forget people's names	.51	.49	*.04*											.52	*.04*
16	forget where he/she is	.38	.57	*.04*											.24	*.06*
17	forget what day it is	.55	.61	*.03*											.42	*.04*
18	thoughts muddled	.68	.72	*.03*											.40	*.04*
19	difficulty deciding	.61	.70	*.03*											.36	*.04*
20	making self understood	.47	.61	*.04*											.33	*.05*
21	keeping clean	.82	.56	*.05*					.71	*.04*						
22	keeping looking nice	.82	.56	*.04*					.71	*.04*						
23	get things from shops	.54	.63	*.04*							.38	*.06*				
24	using money to pay	.97	.61	*.04*							.77	*.08*				
25	looking after finances	.62	.60	*.04*							.50	*.06*				
26	things take longer	.45	.67	*.03*												
27	get in touch with people	.54	.66	*.04*									.32	*.06*		
28	not enough company	.44	.62	*.03*									.22	*.06*		
29	not being able to help	.87	.49	*.04*									.79	*.08*		
30	not playing a useful part	.61	.57	*.04*									.53	*.06*		
31	his/her physical health	.25	.50	*.04*												
		ω_h	.88		.69		.35		.56		.39		.33		.34	

Model fit from non-bootstrapped results:
χ^2 = 1647.018 (df = 406), RMSEA = **.058** (90% CI = .055 - .061), CFI = **.932**
h^2: communalities
ω_h: Omega hierarchical coefficient

Table S4 Bifactor CFA (Model 1a) for **Spanish** version of 31-item **DEMQOL-Proxy** (standardised factor loadings with bootstrapped standard errors)

Qn	DEMQOL-Proxy (n = 909)	h^2	**GEN**	*SE*	**POS**	*SE*	**NEG**	*SE*	**APP**	*SE*	**FIN**	*SE*	**SOC**	*SE*	**COG**	*SE*
1	cheerful	.67	*-.20*	*.06*	.79	*.03*										
2	worried or anxious	.57	.48	*.05*			.58	*.05*								
3	frustrated	.56	.37	*.06*			.65	*.06*								
4	full of energy	.49	*-.17*	*.06*	.68	*.04*										
5	sad	.54	.25	*.06*			.69	*.06*								
6	content	.74	*-.16*	*.06*	.85	*.03*										
7	distressed	.44	.53	*.05*			.39	*.07*								
8	lively	.70	*-.24*	*.06*	.80	*.03*										
9	irritable	.26	.20	*.06*			.47	*.07*								
10	fed-up	.28	.40	*.05*			.34	*.06*								
11	things to look forward	.29	*-.42*	*.05*	.33	*.06*										
12	memory in general	.60	.57	*.06*											.52	*.07*
13	forget long ago things	.71	.54	*.06*											.65	*.06*
14	forget recent things	.77	.59	*.06*											.65	*.06*
15	forget people’s names	.80	.60	*.05*											.67	*.05*
16	forget where he/she is	.73	.60	*.06*											.61	*.05*
17	forget what day it is	.69	.59	*.05*											.58	*.05*
18	thoughts muddled	.72	.49	*.07*											.69	*.05*
19	difficulty deciding	.69	.47	*.07*											.68	*.07*
20	making self understood	.59	.52	*.07*											.56	*.07*
21	keeping clean	.89	.60	*.05*					.73	*.04*						
22	keeping looking nice	.94	.64	*.04*					.73	*.04*						
23	get things from shops	.72	.64	*.05*							.56	*.06*				
24	using money to pay	.88	.64	*.05*							.69	*.04*				
25	looking after finances	.85	.62	*.05*							.69	*.04*				
26	things take longer	.50	.70	*.04*												
27	get in touch with people	.57	.74	*.04*									.13	*.09*		
28	not enough company	.40	.60	*.05*									.19	*.10*		
29	not being able to help	.88	.74	*.04*									.57	*.40*		
30	not playing a useful part	.74	.74	*.04*									.44	*.26*		
31	his/her physical health	.56	.75	*.03*												
		ω_h	.82		.77		.54		.55		.47		.16		.53	

Model fit from non-bootstrapped results:
χ^2 = 1133.244 (df = 407), RMSEA = **.060** (90% CI = .056 - .064), CFI = **.950**
h^2: communalities
ω_h: Omega hierarchical coefficient

To see if potential wording effects in POS items was the main issue undermining measurement invariance, we examined configural invariance for the remaining 23 DEMQOL items and 26 DEMQOL-Proxy items. In the UK study sample, the single-group bifactor CFA model for DEMQOL with 23 items (**Model 1b**: GEN, NEG, COG, SOC, LON) and DEMQOL-Proxy with 26 items (**Model 1b**: GEN, NEG, COG, SOC, FIN, APP) showed good model fit (Table S5 and S7). This was also the case for Latin America study sample (Table S6 and S8), but the NEG domain factor in DEMQOL and SOC domain factor in DEMQOL-Proxy showed signs of factor collapse (Chen *et al.*, 2006). As these domain-specific factor loadings did not attain statistical significance in the bootstrapped models, configural invariance could again not be assumed. In the context of bifactor models, Omega hierarchical coefficients (Table S5 – S8) showed that the general HRQL factor was consistently the most reliable source of influence on responses across all items in the DEMQOL and DEMQOL-Proxy. In other words, while less reliable sources of influence on item responses (i.e. domain-specific loadings) differ between the UK and Latin America, the dominant impact of general HRQL on item responses was consistent across both groups.

Mplus syntax for 23 items in **DEMQOL** Model 1b (Table S5 and S6)

```
Title:
single-group bifactor CFA
for 23 DEMQOL items
UK (EL) vs Latin America (ES)

Data: File = p2croy1066.dat ;

Variable:
Names = lang f0 sev
a1 a2 a3 a4 a5 a6 a7 a8 a9 a10
a11 a12 a13 a14 a15 a16 a17 a18 a19 a20
a21 a22 a23 a24 a25 a26 a27 a28
b1 b2 b3 b4 b5 b6 b7 b8 b9 b10
b11 b12 b13 b14 b15 b16 b17 b18 b19 b20
b21 b22 b23 b24 b25 b26 b27 b28 b29 b30
b31 ;

Missing = all (-1234) ;
Usev =
    a2    a4         a7 a8 a9      a11–a28 ;
!a1 a2 a3 a4 a5 a6 a7 a8 a9 a10

Categorical = all  ;

!! single-group CFA
Useobs = lang eq 0  ;

!! multiple-group CFA
!Grouping = lang (0=EL 1=ES) ;

Analysis:
BOOTSTRAP = 5000  ;
```

```
Model:
gen by
     a2*   a4          a7 a8 a9
!a1* a2 a3 a4 a5 a6 a7 a8 a9 a10
a11 a12 a13 a14 a15 a16 a17 a18 a19 a20
a21 a22 a23 a24 a25 a26 a27 a28 ;

!pos by a1* a3 a5 a6 a10 ;
neg by a4* a11 a12 a13 ;
cog by a14* a15 a16 a17 a18 a19 ;
lon by a8* a20 (eq1) ;
soc by a21* a22 a23 a24 a25 a26 ;

!! fix factor variance
 gen@1            neg@1 cog@1 lon@1 soc@1 ;
!gen@1 pos@1 neg@1 cog@1 lon@1 soc@1 ;

!! orthogonality
gen with             neg@0 cog@0 lon@0 soc@0 ;
!gen with pos@0 neg@0 cog@0 lon@0 soc@0 ;
!pos with neg@0 cog@0 lon@0 soc@0 ;
neg with cog@0 lon@0 soc@0 ;
cog with lon@0 soc@0 ;
lon with soc@0 ;

Output:
!sampstat residual modindices;
CINTERVAL (BCBOOTSTRAP);
```

Table S5 Bifactor CFA (Model 1b) for **English** version of 23 **DEMQOL** items (standardised factor loadings with bootstrapped standard errors)

Qn	DEMQOL (n = 867)	h^2	**GEN**	*SE*	**NEG**	*SE*	**COG**	*SE*	**LON**	*SE*	**SOC**	*SE*
2	worried or anxious	.47	.68	*.03*								
4	frustrated	.83	.54	*.04*	.73	*.08*						
7	sad	.44	.66	*.03*								
8	lonely	.80	.52	*.04*					.73	*.03*		
9	distressed	.62	.79	*.03*								
11	irritable	.45	.58	*.04*	.34	*.06*						
12	fed-up	.58	.65	*.03*	.40	*.06*						
13	things to do but couldn't	.30	.44	*.04*	.33	*.05*						
14	forget recent things	.60	.60	*.03*			.49	*.04*				
15	forgetting who people are	.49	.56	*.04*			.42	*.05*				
16	forgetting what day it is	.47	.51	*.04*			.46	*.05*				
17	your thoughts being muddled	.74	.65	*.03*			.56	*.04*				
18	difficulty making decisions	.62	.74	*.03*			.27	*.05*				
19	poor concentration	.61	.66	*.03*			.41	*.04*				
20	not having enough company	.78	.50	*.04*					.73	*.03*		
21	get on with people close	.62	.60	*.04*							.51	*.06*
22	getting affection that you want	.78	.60	*.04*							.65	*.07*
23	people not listening to you	.62	.63	*.04*							.48	*.07*
24	making yourself understood	.49	.63	*.04*							.29	*.08*
25	getting help when you need it	.61	.70	*.04*							.34	*.07*
26	getting to the toilet in time	.36	.51	*.05*							.31	*.08*
27	how you feel in yourself	.58	.76	*.02*								
28	your health overall	.38	.61	*.03*								
		ω_h	.87		.33		.30		.59		.29	

Model fit from non-bootstrapped results:
χ^2 = 793.336 (df = 213), RMSEA = **.056** (90% CI = .052 - .060), CFI = **.946**
h^2: communalities
ω_h: Omega hierarchical coefficient

Table S6 Bifactor CFA (Model 1b) for **Spanish** version of 23 **DEMQOL** items (standardised factor loadings with bootstrapped standard errors)

Qn	DEMQOL (n = 417)	h^2	**GEN**	*SE*	**NEG**	*SE*	**COG**	*SE*	**LON**	*SE*	**SOC**	*SE*
2	worried or anxious	.38	.62	*.05*								
4	frustrated	.43	.63	*.05*	.18^{ns}	*.10*						
7	sad	.39	.63	*.04*								
8	lonely	.59	.60	*.05*					.48	*.06*		
9	distressed	.37	.61	*.05*								
11	irritable	.54	.60	*.05*	.42	*.16*						
12	fed-up	.76	.66	*.05*	.57^{ns}	*.46*						
13	things to do but couldn't	.49	.66	*.04*	.22	*.09*						
14	forget recent things	.69	.60	*.05*			.58	*.06*				
15	forgetting who people are	.78	.66	*.05*			.59	*.06*				
16	forgetting what day it is	.70	.58	*.05*			.60	*.06*				
17	your thoughts being muddled	.79	.72	*.04*			.51	*.05*				
18	difficulty making decisions	.70	.68	*.05*			.50	*.06*				
19	poor concentration	.74	.74	*.04*			.44	*.07*				
20	not having enough company	.77	.73	*.04*					.48	*.06*		
21	get on with people close	.70	.82	*.04*							.15^{ns}	*.09*
22	getting affection that you want	.68	.78	*.04*							.27	*.08*
23	people not listening to you	.86	.75	*.04*							.55	*.07*
24	making yourself understood	.82	.71	*.04*							.56	*.07*
25	getting help when you need it	.74	.79	*.03*							.33	*.07*
26	getting to the toilet in time	.50	.64	*.05*							.30	*.09*
27	how you feel in yourself	.59	.77	*.04*								
28	your health overall	.45	.67	*.04*								
		ω_h	.90		.19		.37		.28		.18	

Model fit from non-bootstrapped results:
χ^2 = 531.840 (df = 213), RMSEA = **.060** (90% CI = .054 - .066), CFI = **.964**
h^2: communalities
ns: not statistically significant
ω_h: Omega hierarchical coefficient

Mplus syntax for 26 items in **DEMQOL-Proxy** Model 1b (Table S7 and S8)

```
Title:
single-group bifactor CFA
for 26 DEMQOL-Proxy items
UK (EL) vs Latin America (ES)

Data: File = p2croy1066.dat ;

Variable:
Names = lang f0 sev
a1 a2 a3 a4 a5 a6 a7 a8 a9 a10
a11 a12 a13 a14 a15 a16 a17 a18 a19 a20
a21 a22 a23 a24 a25 a26 a27 a28
b1 b2 b3 b4 b5 b6 b7 b8 b9 b10
b11 b12 b13 b14 b15 b16 b17 b18 b19 b20
b21 b22 b23 b24 b25 b26 b27 b28 b29 b30
b31 ;

Missing = all (-1234) ;
Usev =
    b2 b3    b5    b7    b9 b10
!b1 b2 b3 b4 b5 b6 b7 b8 b9 b10
     b12 b13 b14 b15 b16 b17 b18 b19 b20
!b11 b12 b13 b14 b15 b16 b17 b18 b19 b20
b21 - b31 ;


Categorical = all  ;

!! single-group CFA
Useobs = lang eq 0  ;

!! multiple-group CFA
!Grouping = lang (0=EL 1=ES) ;

Analysis:
BOOTSTRAP = 5000  ;
```

```
Model:
gen by
    b2* b3    b5    b7    b9 b10
!b1 b2 b3 b4 b5 b6 b7 b8 b9 b10
     b12 b13 b14 b15 b16 b17 b18 b19 b20
!b11 b12 b13 b14 b15 b16 b17 b18 b19 b20
b21 - b31 ;

!pos by b1* b4 b6 b8 b11 ;
neg by b2* b3 b5 b7 b9 b10 ;
app by b21* b22 (eq11) ;
!fin by b23* b24 b25 ;
fin by b23* ;
fin by b24* b25 (eq12) ;
soc by b27* b28 b29 b30 ;
cog by b12* b13 b14 b15 b16 b17 b18 b19 b20 ;

!! fix factor variance
 gen@1          neg@1 app@1 fin@1 soc@1 cog@1 ;
!gen@1 pos@1 neg@1 app@1 fin@1 soc@1 cog@1 ;


!! orthogonality
 gen with           neg@0 app@0 fin@0 soc@0 cog@0 ;
!gen with pos@0 neg@0 app@0 fin@0 soc@0 cog@0 ;
!pos with neg@0 app@0 fin@0 soc@0 cog@0 ;
neg with app@0 fin@0 soc@0 cog@0 ;
app with fin@0 soc@0 cog@0 ;
fin with soc@0 cog@0 ;
soc with cog@0 ;

Output:
!sampstat residual modindices;
CINTERVAL (BCBOOTSTRAP);
```

Table S7 Bifactor CFA (Model 1b) for **English** version of 26 **DEMQOL-Proxy** items (standardised factor loadings with bootstrapped standard errors)

Qn	DEMQOL-Proxy (n = 909)	h^2	**GEN**	*SE*	**NEG**	*SE*	**APP**	*SE*	**FIN**	*SE*	**SOC**	*SE*	**COG**	*SE*
2	worried or anxious	.52	.52	*.03*	.49	*.04*								
3	frustrated	.51	.51	*.03*	.50	*.04*								
5	sad	.61	.53	*.04*	.57	*.04*								
7	distressed	.65	.59	*.04*	.55	*.04*								
9	irritable	.41	.34	*.04*	.54	*.04*								
10	fed-up	.62	.57	*.03*	.54	*.04*								
12	memory in general	.63	.52	*.04*									.60	*.04*
13	forget long ago things	.37	.50	*.05*									.34	*.06*
14	forget recent things	.89	.63	*.03*									.70	*.04*
15	forget people's names	.50	.56	*.04*									.44	*.05*
16	forget where he/she is	.38	.60	*.04*									.16	*.07*
17	forget what day it is	.55	.67	*.03*									.32	*.06*
18	thoughts muddled	.68	.77	*.03*									.30	*.05*
19	difficulty deciding	.62	.75	*.03*									.25	*.05*
20	making self understood	.48	.66	*.03*									.21	*.07*
21	keeping clean	.81	.56	*.05*			.71	*.04*						
22	keeping looking nice	.83	.57	*.04*			.71	*.04*						
23	get things from shops	.56	.64	*.04*					.39	*.06*				
24	using money to pay	.77	.62	*.04*					.62	*.04*				
25	looking after finances	.74	.60	*.04*					.62	*.04*				
26	things take longer	.46	.68	*.03*										
27	get in touch with people	.55	.67	*.03*							.31	*.05*		
28	not enough company	.43	.61	*.03*							.23	*.06*		
29	not being able to help	.85	.50	*.04*							.78	*.08*		
30	not playing a useful part	.61	.56	*.04*							.54	*.06*		
31	his/her physical health	.25	.50	*.04*										
		ω_h	.88		.35		.56		.39		.33		.34	

Model fit from non-bootstrapped results:
χ^2 = 715.923 (df = 277), RMSEA = **.042** (90% CI = .038 - .046), CFI = **.972**
h^2: communalities
ω_h: Omega hierarchical coefficient

Table S8 Bifactor CFA (Model 1b) for **Spanish** version of 26 **DEMQOL-Proxy** items (standardised factor loadings with bootstrapped standard errors)

Qn	DEMQOL-Proxy (n = 495)	h^2	**GEN**	*SE*	**NEG**	*SE*	**APP**	*SE*	**FIN**	*SE*	**SOC**	*SE*	**COG**	*SE*
2	worried or anxious	.57	.49	*.05*	.58	*.05*								
3	frustrated	.56	.39	*.06*	.64	*.06*								
5	sad	.52	.29	*.06*	.66	*.06*								
7	distressed	.43	.54	*.05*	.38	*.07*								
9	irritable	.26	.21	*.06*	.47	*.07*								
10	fed-up	.27	.39	*.05*	.35	*.07*								
12	memory in general	.60	.56	*.06*									.54	*.07*
13	forget long ago things	.71	.53	*.07*									.66	*.06*
14	forget recent things	.78	.58	*.06*									.66	*.06*
15	forget people's names	.80	.61	*.05*									.66	*.05*
16	forget where he/she is	.73	.61	*.06*									.60	*.05*
17	forget what day it is	.68	.60	*.05*									.58	*.05*
18	thoughts muddled	.71	.52	*.07*									.67	*.06*
19	difficulty deciding	.68	.49	*.08*									.67	*.08*
20	making self understood	.58	.54	*.07*									.54	*.07*
21	keeping clean	.89	.58	*.05*			.75	*.03*						
22	keeping looking nice	.94	.62	*.04*			.75	*.03*						
23	get things from shops	.72	.63	*.05*					.57	*.06*				
24	using money to pay	.88	.63	*.05*					.69	*.04*				
25	looking after finances	.85	.61	*.05*					.69	*.04*				
26	things take longer	.50	.71	*.04*										
27	get in touch with people	.57	.74	*.04*							.12^{ns}	*.10*		
28	not enough company	.40	.61	*.05*							.17^{ns}	*.10*		
29	not being able to help	.90	.75	*.04*							.58^{ns}	*.38*		
30	not playing a useful part	.73	.75	*.04*							.42^{ns}	*.36*		
31	his/her physical health	.55	.74	*.03*										
		ω_h	.89		.52		.58		.48		.15		.53	

Model fit from non-bootstrapped results:
χ^2 = 912.963(df = 277), RMSEA = **.068** (90% CI = .063 - .073), CFI = **.951**
h^2: communalities
ns: not statistically significant
ω_h: Omega hierarchical coefficient

Given indications of factor collapse and weak subscale reliability in Model 1b, we decided to fit a DEMQOL bifactor model without a NEG domain factor (**Model 1c**: GEN, COG, SOC, LON) in both the UK and Latin America study sample (Table S9 and S10). For DEMQOL-Proxy, we fit a bifactor model without a SOC domain factor (**Model 1c**: GEN, NEG, COG, FIN, APP) in both study samples (Table S11 and S12). Model 1c for DEMQOL postulates that "negative emotion" item responses form a core component in self-appraisal of general HRQL. Model 1c for DEMQOL-Proxy postulates that "worries about social functioning" item responses form a core component in informant appraisal of general HRQL. Single-group CFA models showed that these assumptions about general HRQL appraisal were tenable for self- and informant-report in the UK and Latin America.

Mplus syntax for 23 items in **DEMQOL** Model 1c (Table S9 and S10)

```
Title:
single-group bifactor CFA
for 23 DEMQOL items
UK (EL) vs Latin America (ES)

Data: File = p2croy1066.dat ;

Variable:
Names = lang f0 sev
a1 a2 a3 a4 a5 a6 a7 a8 a9 a10
a11 a12 a13 a14 a15 a16 a17 a18 a19 a20
a21 a22 a23 a24 a25 a26 a27 a28
b1 b2 b3 b4 b5 b6 b7 b8 b9 b10
b11 b12 b13 b14 b15 b16 b17 b18 b19 b20
b21 b22 b23 b24 b25 b26 b27 b28 b29 b30
b31 ;

Missing = all (-1234) ;
Usev =
     a2     a4          a7 a8 a9      a11–a28 ;
!a1 a2 a3 a4 a5 a6 a7 a8 a9 a10

Categorical = all  ;

!! single-group CFA
Useobs = lang eq 0  ;

!! multiple-group CFA
!Grouping = lang (0=EL 1=ES) ;

Analysis:
BOOTSTRAP = 5000  ;
```

```
Model:
gen by
       a2*    a4          a7 a8 a9
!a1* a2 a3 a4 a5 a6 a7 a8 a9 a10
a11 a12 a13 a14 a15 a16 a17 a18 a19 a20
a21 a22 a23 a24 a25 a26 a27 a28 ;

!pos by a1* a3 a5 a6 a10 ;
!neg by a4* a11 a12 a13 ;
cog by a14* a15 a16 a17 a18 a19 ;
lon by a8* a20 (eq1) ;
soc by a21* a22 a23 a24 a25 a26 ;

!! fix factor variance
 gen@1                    cog@1 lon@1 soc@1 ;
!gen@1 pos@1 neg@1 cog@1 lon@1 soc@1 ;

!! orthogonality
gen with                    cog@0 lon@0 soc@0 ;
!gen with pos@0 neg@0 cog@0 lon@0 soc@0 ;
!pos with neg@0 cog@0 lon@0 soc@0 ;
!neg with cog@0 lon@0 soc@0 ;
cog with lon@0 soc@0 ;
lon with soc@0 ;

Output:
!sampstat residual modindices;
CINTERVAL (BCBOOTSTRAP);
```

Table S9 Bifactor CFA (Model 1c) for **English** version of 23 **DEMQOL** items (standardised factor loadings with bootstrapped standard errors)

Qn	DEMQOL (n = 867)	h^2	**GEN**	*SE*	**COG**	*SE*	**LON**	*SE*	**SOC**	*SE*
2	worried or anxious	.46	.68	*.03*						
4	frustrated	.40	.63	*.03*						
7	sad	.43	.66	*.03*						
8	lonely	.80	.52	*.04*			.73	*.03*		
9	distressed	.61	.78	*.03*						
11	irritable	.39	.62	*.03*						
12	fed-up	.49	.70	*.03*						
13	things to do but couldn't	.24	.49	*.04*						
14	forget recent things	.60	.59	*.03*	.50	*.04*				
15	forgetting who people are	.49	.55	*.04*	.44	*.05*				
16	forgetting what day it is	.47	.50	*.04*	.47	*.05*				
17	your thoughts being muddled	.74	.64	*.03*	.57	*.04*				
18	difficulty making decisions	.61	.72	*.03*	.30	*.05*				
19	poor concentration	.61	.65	*.03*	.43	*.04*				
20	not having enough company	.78	.50	*.04*			.73	*.03*		
21	get on with people close	.62	.60	*.04*					.51	*.06*
22	getting affection that you want	.78	.59	*.04*					.66	*.06*
23	people not listening to you	.63	.62	*.04*					.49	*.07*
24	making yourself understood	.49	.63	*.04*					.31	*.07*
25	getting help when you need it	.61	.70	*.04*					.35	*.07*
26	getting to the toilet in time	.36	.50	*.05*					.33	*.08*
27	how you feel in yourself	.57	.76	*.02*						
28	your health overall	.37	.61	*.03*						
		ω_h	.88		.32		.60		.31	

Model fit from non-bootstrapped results:
χ^2 = 664.715 (df = 217), RMSEA = **.064** (90% CI = .060 - .068), CFI = **.928**
h^2: communalities
ω_h: Omega hierarchical coefficient

Table S10 Bifactor CFA (Model 1c) for **Spanish** version of 23 **DEMQOL** items (standardised factor loadings with bootstrapped standard errors)

Qn	DEMQOL (n = 417)	h^2	**GEN**	*SE*	**COG**	*SE*	**LON**	*SE*	**SOC**	*SE*
2	worried or anxious	.38	.61	*.05*						
4	frustrated	.41	.64	*.05*						
7	sad	.39	.63	*.04*						
8	lonely	.59	.60	*.05*			.49	*.05*		
9	distressed	.37	.61	*.05*						
11	irritable	.42	.64	*.05*						
12	fed-up	.49	.70	*.04*						
13	things to do but couldn't	.46	.68	*.04*						
14	forget recent things	.69	.60	*.05*	.58	*.06*				
15	forgetting who people are	.78	.65	*.05*	.59	*.06*				
16	forgetting what day it is	.70	.58	*.05*	.60	*.05*				
17	your thoughts being muddled	.79	.72	*.04*	.52	*.05*				
18	difficulty making decisions	.71	.67	*.05*	.50	*.06*				
19	poor concentration	.74	.74	*.04*	.44	*.06*				
20	not having enough company	.77	.73	*.04*			.49	*.05*		
21	get on with people close	.70	.82	*.04*					.17	*.09*
22	getting affection that you want	.68	.77	*.04*					.28	*.08*
23	people not listening to you	.87	.75	*.04*					.55	*.07*
24	making yourself understood	.82	.71	*.04*					.56	*.07*
25	getting help when you need it	.73	.79	*.03*					.34	*.07*
26	getting to the toilet in time	.50	.64	*.05*					.31	*.09*
27	how you feel in yourself	.59	.77	*.04*						
28	your health overall	.44	.67	*.04*						
		ω_h	.91		.38		.28		.19	

Model fit from non-bootstrapped results:
χ^2 = 568.640 (df = 217), RMSEA = **.062** (90% CI = .056 - .069), CFI = **.961**
h^2: communalities
ω_h: Omega hierarchical coefficient

Mplus syntax for 26 items in **DEMQOL-Proxy** Model 1c (Table S11 and S12)

```
Title:
single-group bifactor CFA
for 26 DEMQOL-Proxy items
UK (EL) vs Latin America (ES)

Data: File = p2croy1066.dat ;

Variable:
Names = lang f0 sev
a1 a2 a3 a4 a5 a6 a7 a8 a9 a10
a11 a12 a13 a14 a15 a16 a17 a18 a19 a20
a21 a22 a23 a24 a25 a26 a27 a28
b1 b2 b3 b4 b5 b6 b7 b8 b9 b10
b11 b12 b13 b14 b15 b16 b17 b18 b19 b20
b21 b22 b23 b24 b25 b26 b27 b28 b29 b30
b31 ;

Missing = all (-1234) ;
Usev =
     b2 b3      b5      b7      b9 b10
!b1 b2 b3 b4 b5 b6 b7 b8 b9 b10
        b12 b13 b14 b15 b16 b17 b18 b19 b20
!b11 b12 b13 b14 b15 b16 b17 b18 b19 b20
b21 - b31 ;


Categorical = all  ;

!! single-group CFA
Useobs = lang eq 0  ;

!! multiple-group CFA
!Grouping = lang (0=EL 1=ES) ;

Analysis:
BOOTSTRAP = 5000  ;
```

```
Model:
gen by
     b2* b3      b5      b7      b9 b10
!b1 b2 b3 b4 b5 b6 b7 b8 b9 b10
        b12 b13 b14 b15 b16 b17 b18 b19 b20
!b11 b12 b13 b14 b15 b16 b17 b18 b19 b20
b21 - b31 ;

!pos by b1* b4 b6 b8 b11 ;
neg by b2* b3 b5 b7 b9 b10 ;
app by b21* b22 (eq11) ;
!fin by b23* b24 b25 ;
fin by b23* ;
fin by b24* b25 (eq12) ;
!soc by b27* b28 b29 b30 ;
cog by b12* b13 b14 b15 b16 b17 b18 b19 b20 ;

!! fix factor variance
 gen@1          neg@1 app@1 fin@1            cog@1 ;
!gen@1 pos@1 neg@1 app@1 fin@1 soc@1 cog@1 ;


!! orthogonality
 gen with            neg@0 app@0 fin@0            cog@0 ;
!gen with pos@0 neg@0 app@0 fin@0 soc@0 cog@0 ;
!pos with neg@0 app@0 fin@0 soc@0 cog@0 ;
neg with app@0 fin@0  cog@0 ;
app with fin@0  cog@0 ;
fin with cog@0 ;
!soc with cog@0 ;

Output:
!sampstat residual modindices;
CINTERVAL (BCBOOTSTRAP);
```

Table S11 Bifactor CFA (Model 1c) for **English** version of 26 **DEMQOL-Proxy** items (standardised factor loadings with bootstrapped standard errors)

Qn	DEMQOL-Proxy (n = 909)	h^2	**GEN**	*SE*	**NEG**	*SE*	**APP**	*SE*	**FIN**	*SE*	**COG**	*SE*
2	worried or anxious	.52	.52	*.03*	.50	*.04*						
3	frustrated	.51	.50	*.03*	.51	*.04*						
5	sad	.61	.52	*.04*	.58	*.04*						
7	distressed	.65	.59	*.04*	.56	*.04*						
9	irritable	.41	.34	*.04*	.54	*.04*						
10	fed-up	.62	.57	*.03*	.54	*.04*						
12	memory in general	.63	.51	*.04*							.61	*.04*
13	forget long ago things	.37	.49	*.05*							.37	*.05*
14	forget recent things	.87	.61	*.03*							.71	*.03*
15	forget people's names	.51	.54	*.04*							.47	*.05*
16	forget where he/she is	.38	.58	*.04*							.20	*.07*
17	forget what day it is	.55	.64	*.03*							.36	*.05*
18	thoughts muddled	.68	.75	*.03*							.34	*.05*
19	difficulty deciding	.61	.73	*.03*							.29	*.05*
20	making self understood	.48	.64	*.03*							.26	*.06*
21	keeping clean	.81	.55	*.05*			.71	*.03*				
22	keeping looking nice	.83	.57	*.04*			.71	*.03*				
23	get things from shops	.56	.64	*.04*					.39	*.06*		
24	using money to pay	.77	.62	*.04*					.62	*.03*		
25	looking after finances	.74	.59	*.04*					.62	*.03*		
26	things take longer	.46	.68	*.03*								
27	get in touch with people	.50	.71	*.03*								
28	not enough company	.41	.64	*.03*								
29	not being able to help	.39	.62	*.03*								
30	not playing a useful part	.43	.66	*.03*								
31	his/her physical health	.25	.50	*.04*								
		ω_h	.90		.46		.56		.38		.28	

Model fit from non-bootstrapped results:
χ^2 = 1000.566 (df = 281), RMSEA = **.053** (90% CI = .050 - .057), CFI = **.954**
h^2: communalities
ω_h: Omega hierarchical coefficient

Table S12 Bifactor CFA (Model 1c) for **Spanish** version of 26 **DEMQOL-Proxy** items (standardised factor loadings with bootstrapped standard errors)

Qn	DEMQOL-Proxy (n = 495)	h^2	**GEN**	*SE*	**NEG**	*SE*	**APP**	*SE*	**FIN**	*SE*	**COG**	*SE*
2	worried or anxious	.57	.48	*.05*	.59	*.05*						
3	frustrated	.56	.38	*.06*	.65	*.06*						
5	sad	.52	.28	*.06*	.66	*.06*						
7	distressed	.43	.53	*.05*	.38	*.07*						
9	irritable	.26	.20	*.06*	.47	*.07*						
10	fed-up	.27	.39	*.05*	.35	*.06*						
12	memory in general	.60	.55	*.06*							.55	*.06*
13	forget long ago things	.71	.52	*.06*							.67	*.06*
14	forget recent things	.78	.57	*.06*							.67	*.05*
15	forget people's names	.80	.60	*.05*							.67	*.05*
16	forget where he/she is	.73	.59	*.06*							.62	*.05*
17	forget what day it is	.68	.58	*.05*							.59	*.05*
18	thoughts muddled	.71	.51	*.06*							.67	*.05*
19	difficulty deciding	.68	.48	*.07*							.67	*.07*
20	making self understood	.58	.53	*.06*							.55	*.07*
21	keeping clean	.89	.58	*.05*			.75	*.03*				
22	keeping looking nice	.94	.62	*.04*			.75	*.03*				
23	get things from shops	.72	.62	*.05*					.57	*.06*		
24	using money to pay	.88	.63	*.05*					.70	*.04*		
25	looking after finances	.85	.61	*.05*					.70	*.04*		
26	things take longer	.49	.70	*.04*								
27	get in touch with people	.57	.75	*.03*								
28	not enough company	.40	.63	*.04*								
29	not being able to help	.69	.83	*.03*								
30	not playing a useful part	.68	.82	*.03*								
31	his/her physical health	.53	.73	*.03*								
		ω_h	.90		.53		.59		.49		.54	

Model fit from non-bootstrapped results:
χ^2 = 944.623 (df = 281), RMSEA = **.069** (90% CI = .064 - .074), CFI = **.948**
h^2: communalities
ω_h: Omega hierarchical coefficient

Based on Model 1c, the multiple-group CFA **Model 2a** offered direct statistical testing of measurement invariance between the two study samples. The results showed good model fit when we hypothesised that there was configural invariance between English and Spanish versions of 23 DEMQOL items (Table S13 and S14). This was also the case for **Model 2a** of the 26 DEMQOL-Proxy items (Table S15 and S16).

Mplus syntax for 23 items in **DEMQOL** Model 2a (Table S13 and S14)

```
Title:
multiple-group bifactor CFA
CONFIGURAL INV for 23 DEMQOL items
UK (EL) vs Latin America (ES)

Data: File = p2croy1066.dat ;

Variable:
Names = lang f0 sev
a1 a2 a3 a4 a5 a6 a7 a8 a9 a10
a11 a12 a13 a14 a15 a16 a17 a18 a19 a20
a21 a22 a23 a24 a25 a26 a27 a28
b1 b2 b3 b4 b5 b6 b7 b8 b9 b10
b11 b12 b13 b14 b15 b16 b17 b18 b19 b20
b21 b22 b23 b24 b25 b26 b27 b28 b29 b30
b31 ;

Missing = all (-1234) ;
Usev =
   a2    a4       a7 a8 a9     a11–a28 ;
!a1 a2 a3 a4 a5 a6 a7 a8 a9 a10

Categorical = all  ;

!! multiple-group CFA
Grouping = lang (0=EL 1=ES) ;

Analysis:
BOOTSTRAP = 5000  ;

Model:
gen by
  a2*   a4     a7 a8 a9
a11 a12@1 a13 a14 a15 a16 a17 a18 a19 a20
a21 a22 a23 a24 a25 a26 a27 a28 ;

!pos by a1* a3 a5 a6 a10 ;
!neg by a4* a11 a12 a13 ;
cog by a14* a15 a16@1 a17 a18 a19 ;
lon by a8@1 a20@1 ;
soc by a21* a22 a23@1 a24 a25 a26 ;

!! orthogonality
gen with                cog@0 lon@0 soc@0 ;
cog with lon@0 soc@0 ;
lon with soc@0 ;

Model ES:

!! free factor loadings
gen by
  a2*   a4     a7 a8 a9
a11 a12@1 a13 a14 a15 a16 a17 a18 a19 a20
a21 a22 a23 a24 a25 a26 a27 a28 ;

!pos by a1* a3 a5 a6 a10 ;
!neg by a4* a11 a12 a13 ;
cog by a14* a15 a16@1 a17 a18 a19 ;
lon by a8@1 a20@1 ;
soc by a21* a22 a23@1 a24 a25 a26 ;

!! free thresholds
[ a2$1 a2$2 a2$3 ] ;
[ a4$1 a4$2 a4$3 ] ;
[ a7$1 a7$2 a7$3 ] ;
[ a8$1 a8$2 a8$3 ] ;
[ a9$1 a9$2 a9$3 ] ;
[ a11$1 a11$2 a11$3 ] ;
[ a12$1 a12$2 a12$3 ] ;
[ a13$1 a13$2 a13$3 ] ;
[ a14$1 a14$2 a14$3 ] ;
[ a15$1 a15$2 a15$3 ] ;
[ a16$1 a16$2 a16$3 ] ;
[ a17$1 a17$2 a17$3 ] ;
[ a18$1 a18$2 a18$3 ] ;
[ a19$1 a19$2 a19$3 ] ;
[ a20$1 a20$2 a20$3 ] ;
[ a21$1 a21$2 a21$3 ] ;
[ a22$1 a22$2 a22$3 ] ;
[ a23$1 a23$2 a23$3 ] ;
[ a24$1 a24$2 a24$3 ] ;
[ a25$1 a25$2 a25$3 ] ;
[ a26$1 a26$2 a26$3 ] ;
[ a27$1 a27$2 a27$3 ] ;
[ a28$1 a28$2 a28$3 ] ;

!! fix scale factors to 1
{ a2@1  a4@1    a7@1 a8@1 a9@1 a11-a28@1 } ;

!! fix factor means to 0
[ gen@0        cog@0 lon@0 soc@0] ;

Output:
!sampstat residual modindices;
CINTERVAL (BCBOOTSTRAP);

!Savedata: DIFFTEST = strict.dat ;
```

Table S13 Multiple-group CFA (Model 2a) for 23 **DEMQOL** items (*unstandardised* factor loadings with bootstrapped standard errors): configural invariance estimates for **UK** study sample

Qn	DEMQOL (n = 1284)<br>**EL (n = 867)**<br>ES (n = 417)	h^2	**GEN**	*SE*	**COG**	*SE*	**LON**	*SE*	**SOC**	*SE*
2	worried or anxious	.46	.97	*.05*						
4	frustrated	.40	.90	*.04*						
7	sad	.43	.94	*.05*						
8	lonely	.80	.74	*.06*			**1.00**	**		
9	distressed	.61	1.12	*.05*						
11	irritable	.39	.89	*.05*						
12	fed-up	.49	**1.00**	**						
13	things to do but couldn't	.24	.69	*.05*						
14	forget recent things	.60	.84	*.05*	1.05	*.13*				
15	forgetting who people are	.49	.78	*.06*	.92	*.13*				
16	forgetting what day it is	.47	.71	*.06*	**1.00**	**				
17	your thoughts being muddled	.74	.91	*.05*	1.21	*.15*				
18	difficulty making decisions	.61	1.03	*.05*	.62	*.13*				
19	poor concentration	.61	.93	*.05*	.91	*.14*				
20	not having enough company	.78	.71	*.06*			**1.00**	**		
21	get on with people close	.62	.85	*.06*					1.04	*.23*
22	getting affection that you want	.78	.84	*.07*					1.33	*.32*
23	people not listening to you	.63	.89	*.07*					**1.00**	**
24	making yourself understood	.49	.89	*.06*					.62	*.11*
25	getting help when you need it	.61	.99	*.06*					.72	*.12*
26	getting to the toilet in time	.36	.71	*.08*					.66	*.16*
27	how you feel in yourself	.57	1.08	*.05*						
28	your health overall	.37	.87	*.06*						
	Factor variance (EL)		.49	*.04*	.22	*.04*	.54	*.04*	.24	*.07*

Model fit from non-bootstrapped results:
χ^2 = 1553.474 (df = 434), English (EL) χ^2 = 1010.865, Spanish (ES) χ^2 = 542.609
RMSEA = **.063** (90% CI = .060 - .067), CFI = **.943**
h^2: communalities
** unstandardised factor loading fixed at value of 1

Table S14 Multiple-group CFA (Model 2a) for 23 **DEMQOL** items (*unstandardised* factor loadings with bootstrapped standard errors): configural invariance estimates for **Latin America** study sample

Qn	DEMQOL (n = 1284)<br>EL (n = 867)<br>**ES (n = 417)**	h^2	**GEN**	*SE*	**COG**	*SE*	**LON**	*SE*	**SOC**	*SE*
2	worried or anxious	.38	.88	*.08*						
4	frustrated	.41	.92	*.07*						
7	sad	.39	.89	*.08*						
8	lonely	.59	.85	*.07*			**1.00**	**		
9	distressed	.37	.87	*.08*						
11	irritable	.42	.92	*.07*						
12	fed-up	.49	**1.00**	**						
13	things to do but couldn't	.46	.97	*.07*						
14	forget recent things	.69	.85	*.09*	.96	*.12*				
15	forgetting who people are	.78	.93	*.09*	.99	*.11*				
16	forgetting what day it is	.70	.83	*.09*	**1.00**	**				
17	your thoughts being muddled	.79	1.03	*.08*	.85	*.09*				
18	difficulty making decisions	.71	.96	*.09*	.84	*.12*				
19	poor concentration	.74	1.05	*.09*	.73	*.12*				
20	not having enough company	.77	1.04	*.08*			**1.00**	**		
21	get on with people close	.70	1.17	*.08*					.30	*.15*
22	getting affection that you want	.68	1.10	*.09*					.51	*.13*
23	people not listening to you	.87	1.07	*.08*					**1.00**	**
24	making yourself understood	.82	1.01	*.08*					1.02	*.20*
25	getting help when you need it	.73	1.12	*.08*					.62	*.14*
26	getting to the toilet in time	.50	.91	*.09*					.56	*.17*
27	how you feel in yourself	.59	1.09	*.08*						
28	your health overall	.44	.95	*.08*						
	Factor variance (ES)		.49	*.06*	.36	*.07*	.24	*.05*	.31	*.07*

Model fit from non-bootstrapped results:
χ^2 = 1553.474 (df = 434), English (EL) χ^2 = 1010.865, Spanish (ES) χ^2 = 542.609
RMSEA = **.063** (90% CI = .060 - .067), CFI = **.943**
h^2: communalities
** unstandardised factor loading fixed at value of 1

Mplus syntax for 26 items in **DEMQOL-Proxy** Model 2a (Table S15 and S16)

```
Title:
multiple-group bifactor CFA
CONFIGURAL INV for 26 DEMQOL-Proxy items
UK (EL) vs Latin America (ES)

Data: File = p2croy1066.dat ;

Variable:
Names = lang f0 sev
a1 a2 a3 a4 a5 a6 a7 a8 a9 a10
a11 a12 a13 a14 a15 a16 a17 a18 a19 a20
a21 a22 a23 a24 a25 a26 a27 a28
b1 b2 b3 b4 b5 b6 b7 b8 b9 b10
b11 b12 b13 b14 b15 b16 b17 b18 b19 b20
b21 b22 b23 b24 b25 b26 b27 b28 b29 b30
b31 ;

Missing = all (-1234) ;
Usev =
    b2 b3     b5     b7     b9 b10
!b1 b2 b3 b4 b5 b6 b7 b8 b9 b10
     b12 b13 b14 b15 b16 b17 b18 b19 b20
!b11 b12 b13 b14 b15 b16 b17 b18 b19 b20
b21 - b31 ;

Categorical = all  ;

!! multiple-group CFA
Grouping = lang (0=EL 1=ES) ;

Analysis:
BOOTSTRAP = 5000  ;

Model:
gen by
    b2* b3   b5    b7    b9 b10
!b1* b2 b3 b4 b5 b6 b7 b8 b9 b10
b12 b13 b14 b15 b16 b17 b18 b19 b20
!b11 b12 b13 b14 b15 b16 b17 b18 b19 b20
b21 b22 b23 b24 b25 b26 b27@1 b28 b29 b30
b31 ;

!pos by b1* b4 b6 b8 b11 ;
neg by b2* b3 b5@1 b7 b9 b10 ;
app by b21@1 b22@1 ;
!fin by b23* b24 b25 ;
fin by b23@1 ;
fin by b24* b25 (eq12) ;
!soc by b27* b28 b29 b30 ;
cog by b12* b13 b14@1 b15 b16 b17 b18 b19 b20 ;

!! orthogonality
gen with     neg@0 app@0 fin@0    cog@0 ;
neg with app@0 fin@0  cog@0 ;
app with fin@0  cog@0 ;
fin with cog@0 ;

Model ES:
!! free factor loadings
gen by
    b2* b3   b5    b7    b9 b10
!b1* b2 b3 b4 b5 b6 b7 b8 b9 b10
b12 b13 b14 b15 b16 b17 b18 b19 b20
!b11 b12 b13 b14 b15 b16 b17 b18 b19 b20
b21 b22 b23 b24 b25 b26 b27@1 b28 b29 b30
b31 ;

!pos by b1* b4 b6 b8 b11 ;
neg by b2* b3 b5@1 b7 b9 b10 ;
app by b21@1 b22@1 ;
!fin by b23* b24 b25 ;
fin by b23@1 ;
fin by b24* b25 (eq22) ;
!soc by b27* b28 b29 b30 ;
cog by b12* b13 b14@1 b15 b16 b17 b18 b19 b20 ;

!! free thresholds
[ b2$1 b2$2 b2$3 ] ;
[ b3$1 b3$2 b3$3 ] ;
[ b5$1 b5$2 b5$3 ] ;
[ b7$1 b7$2 b7$3 ] ;
[ b9$1 b9$2 b9$3 ] ;
[ b10$1 b10$2 b10$3 ] ;
[ b12$1 b12$2 b12$3 ] ;
[ b13$1 b13$2 b13$3 ] ;
[ b14$1 b14$2 b14$3 ] ;
[ b15$1 b15$2 b15$3 ] ;
[ b16$1 b16$2 b16$3 ] ;
[ b17$1 b17$2 b17$3 ] ;
[ b18$1 b18$2 b18$3 ] ;
[ b19$1 b19$2 b19$3 ] ;
[ b20$1 b20$2 b20$3 ] ;
[ b21$1 b21$2 b21$3 ] ;
[ b22$1 b22$2 b22$3 ] ;
[ b23$1 b23$2 b23$3 ] ;
[ b24$1 b24$2 b24$3 ] ;
[ b25$1 b25$2 b25$3 ] ;
[ b26$1 b26$2 b26$3 ] ;
[ b27$1 b27$2 b27$3 ] ;
[ b28$1 b28$2 b28$3 ] ;
[ b29$1 b29$2 b29$3 ] ;
[ b30$1 b30$2 b30$3 ] ;
[ b31$1 b31$2 b31$3 ] ;

!! fix scale factors to 1
{ b2@1 b3@1 b5@1 b7@1 b9@1 b10@1 b12-b31@1 } ;

!! fix factor means to 0
[ gen@0   neg@0 app@0 fin@0    cog@0] ;

Output:
!sampstat residual modindices;
CINTERVAL (BCBOOTSTRAP);

!Savedata: DIFFTEST = strict.dat ;
```

Table S15 Multiple-group CFA (Model 2a) for 26 **DEMQOL-Proxy** items (*unstandardised* factor loadings with bootstrapped standard errors): configural invariance estimates for **UK** study sample

Qn	DEMQOL (n = 1404) **EL (n = 909)** ES (n = 495)	h^2	**GEN**	*SE*	**NEG**	*SE*	**APP**	*SE*	**FIN**	*SE*	**COG**	*SE*
2	worried or anxious	.52	.73	*.06*	**1.00**	**						
3	frustrated	.51	.71	*.06*	1.02	*.12*						
5	sad	.61	.74	*.06*	1.17	*.11*						
7	distressed	.65	.83	*.07*	1.12	*.10*						
9	irritable	.41	.48	*.06*	1.09	*.15*						
10	fed-up	.62	.80	*.06*	1.10	*.12*						
12	memory in general	.63	.71	*.06*							**1.00**	**
13	forget long ago things	.37	.68	*.07*							.60	*.09*
14	forget recent things	.87	.86	*.06*							1.16	*.08*
15	forget people's names	.51	.76	*.06*							.77	*.09*
16	forget where he/she is	.38	.82	*.07*							.33	*.11*
17	forget what day it is	.55	.91	*.06*							.60	*.09*
18	thoughts muddled	.68	1.05	*.06*							.56	*.08*
19	difficulty deciding	.61	1.02	*.06*							.48	*.08*
20	making self understood	.48	.90	*.06*							.43	*.11*
21	keeping clean	.81	.78	*.08*			**1.00**	**				
22	keeping looking nice	.83	.80	*.07*			**1.00**	**				
23	get things from shops	.61	.89	*.06*					**1.00**	**		
24	using money to pay	.75	.87	*.06*					1.30	*.10*		
25	looking after finances	.72	.84	*.06*					1.30	*.10*		
26	things take longer	.46	.95	*.05*								
27	get in touch with people	.50	**1.00**	**								
28	not enough company	.41	.90	*.05*								
29	not being able to help	.39	.88	*.05*								
30	not playing a useful part	.43	.92	*.05*								
31	his/her physical health	.25	.70	*.06*								
	Factor variance (EL)		.50	*.04*	.25	*.04*	.51	*.05*	.21	*.03*	.37	*.04*

Model fit from non-bootstrapped results:
χ^2 = 1930.082 (df = 562), English (EL) χ^2 = 909.674, Spanish (ES) χ^2 = 1020.408
RMSEA = **.059** (90% CI = .056 - .062), CFI = **.952**
h^2: communalities
** unstandardised factor loading fixed at value of 1
ns: not statistically significant

Table S16 Multiple-group CFA (Model 2a) for 26 **DEMQOL-Proxy** items (*unstandardised* factor loadings with bootstrapped standard errors): configural invariance estimates for **Latin America** study sample

Qn	DEMQOL (n = 1404) EL (n = 909) **ES (n = 495)**	h^2	**GEN**	*SE*	**NEG**	*SE*	**APP**	*SE*	**FIN**	*SE*	**COG**	*SE*
2	worried or anxious	.57	.64	*.08*	**1.00**	**						
3	frustrated	.56	.51	*.09*	1.11	*.13*						
5	sad	.52	.38	*.08*	1.13	*.15*						
7	distressed	.43	.71	*.08*	.66	*.13*						
9	irritable	.26	.27	*.08*	.80	*.13*						
10	fed-up	.27	.51	*.07*	.60	*.13*						
12	memory in general	.60	.73	*.09*							**1.00**	**
13	forget long ago things	.71	.69	*.09*							1.23	*.12*
14	forget recent things	.78	.76	*.08*							1.23	*.11*
15	forget people's names	.80	.79	*.08*							1.22	*.15*
16	forget where he/she is	.73	.79	*.08*							1.13	*.15*
17	forget what day it is	.68	.78	*.07*							1.08	*.14*
18	thoughts muddled	.71	.67	*.09*							1.23	*.22*
19	difficulty deciding	.68	.64	*.10*							1.23	*.25*
20	making self understood	.58	.71	*.09*							1.01	*.22*
21	keeping clean	.89	.77	*.07*			**1.00**	**				
22	keeping looking nice	.94	.82	*.07*			**1.00**	**				
23	get things from shops	.69	.84	*.07*					**1.00**	**		
24	using money to pay	.89	.83	*.07*					1.30	*.10*		
25	looking after finances	.86	.81	*.07*					1.30	*.10*		
26	things take longer	.49	.93	*.06*								
27	get in touch with people	.57	**1.00**	**								
28	not enough company	.40	.84	*.06*								
29	not being able to help	.69	1.10	*.06*								
30	not playing a useful part	.68	1.10	*.06*								
31	his/her physical health	.53	.97	*.06*								
	Factor variance (ES)		.57	*.05*	.34	*.06*	.56	*.05*	.29	*.06*	.30	*.07*

Model fit from non-bootstrapped results:
χ^2 = 1930.082 (df = 562), English (EL) χ^2 = 909.674, Spanish (ES) χ^2 = 1020.408
RMSEA = **.059** (90% CI = .056 - .062), CFI = **.952**
h^2: communalities
** unstandardised factor loading fixed at value of 1
ns: not statistically significant

Mplus syntax for 23 items in **DEMQOL** Model 2b (Table 2 in main paper)

```
Title:
multiple-group bifactor CFA
STRONG INV for 23 DEMQOL items
UK (EL) vs Latin America (ES)

Data: File = p2croy1066.dat ;

Variable:
Names = lang f0 sev
a1 a2 a3 a4 a5 a6 a7 a8 a9 a10
a11 a12 a13 a14 a15 a16 a17 a18 a19 a20
a21 a22 a23 a24 a25 a26 a27 a28
b1 b2 b3 b4 b5 b6 b7 b8 b9 b10
b11 b12 b13 b14 b15 b16 b17 b18 b19 b20
b21 b22 b23 b24 b25 b26 b27 b28 b29 b30
b31 ;

Missing = all (-1234) ;
Usev =
    a2    a4        a7 a8 a9      a11–a28 ;
!a1 a2 a3 a4 a5 a6 a7 a8 a9 a10

Categorical = all  ;

!! single-group CFA
!Useobs = lang eq 0  ;

!! multiple-group CFA
Grouping = lang (0=EL 1=ES) ;

Analysis:
!BOOTSTRAP = 5000  ;
DIFFTEST = strict.dat ;
```

```
Model:
gen by
   a2*   a4      a7 a8 a9
a11 a12@1 a13 a14 a15 a16 a17 a18 a19 a20
a21 a22 a23 a24 a25 a26 a27 a28 ;

!pos by a1* a3 a5 a6 a10 ;
!neg by a4* a11 a12 a13 ;
cog by a14* a15 a16@1 a17 a18 a19 ;
lon by a8@1 a20@1 ;
soc by a21* a22 a23@1 a24 a25 a26 ;

!! orthogonality
gen with                  cog@0 lon@0 soc@0 ;
!gen with pos@0 neg@0 cog@0 lon@0 soc@0 ;
!pos with neg@0 cog@0 lon@0 soc@0 ;
!neg with cog@0 lon@0 soc@0 ;
cog with lon@0 soc@0 ;
lon with soc@0 ;

Output:
sampstat residual modindices;
!CINTERVAL (BCBOOTSTRAP);
```

Mplus syntax for 26 items in **DEMQOL-Proxy** Model 2b (Table 3 in main paper)

```
Title:
multiple-group bifactor CFA
STRONG INV for 26 DEMQOL-Proxy items
UK (EL) vs Latin America (ES)

Data: File = p2croy1066.dat ;

Variable:
Names = lang f0 sev
a1 a2 a3 a4 a5 a6 a7 a8 a9 a10
a11 a12 a13 a14 a15 a16 a17 a18 a19 a20
a21 a22 a23 a24 a25 a26 a27 a28
b1 b2 b3 b4 b5 b6 b7 b8 b9 b10
b11 b12 b13 b14 b15 b16 b17 b18 b19 b20
b21 b22 b23 b24 b25 b26 b27 b28 b29 b30
b31 ;

Missing = all (-1234) ;
Usev =
     b2 b3      b5      b7      b9 b10
!b1 b2 b3 b4 b5 b6 b7 b8 b9 b10
       b12 b13 b14 b15 b16 b17 b18 b19 b20
!b11 b12 b13 b14 b15 b16 b17 b18 b19 b20
b21 - b31 ;


Categorical = all  ;

!! single-group CFA
!Useobs = lang eq 0  ;

!! multiple-group CFA
Grouping = lang (0=EL 1=ES) ;

Analysis:
!BOOTSTRAP = 5000  ;
DIFFTEST = strict.dat ;
```

```
Model:
gen by
      b2* b3   b5    b7    b9 b10
!b1* b2 b3 b4 b5 b6 b7 b8 b9 b10
b12 b13 b14 b15 b16 b17 b18 b19 b20
!b11 b12 b13 b14 b15 b16 b17 b18 b19 b20
b21 b22 b23 b24 b25 b26 b27@1 b28 b29 b30
b31 ;

!pos by b1* b4 b6 b8 b11 ;
neg by b2* b3 b5@1 b7 b9 b10 ;
app by b21@1 b22@1 ;
!fin by b23* b24 b25 ;
fin by b23@1 ;
fin by b24* b25 (eq12) ;
!soc by b27* b28 b29 b30 ;
cog by b12* b13 b14@1 b15 b16 b17 b18 b19 b20 ;

!! orthogonality
 gen with           neg@0 app@0 fin@0           cog@0 ;
!gen with pos@0 neg@0 app@0 fin@0 soc@0 cog@0 ;
!pos with neg@0 app@0 fin@0 soc@0 cog@0 ;
neg with app@0 fin@0  cog@0 ;
app with fin@0  cog@0 ;
fin with cog@0 ;
!soc with cog@0 ;

Output:
sampstat residual modindices;
!CINTERVAL (BCBOOTSTRAP);
```

With tenable support for measurement invariance in a subset of DEMQOL and DEMQOL-Proxy items (**Model 2b** reported in Table 1 and 2 in the main paper), we combined both study samples for a single-group CFA (**Model 3**) and added linguistic group as an external covariate predicting the latent means (Table S17 and S18).

Mplus syntax for 23 items in **DEMQOL** Model 3 (Table S17)

Title:<br>combined-group bifactor CFA<br>for 23 DEMQOL items<br><br>Data: File = p2croy1066.dat ;<br><br>Variable:<br>Names = lang f0 sev<br>a1 a2 a3 a4 a5 a6 a7 a8 a9 a10<br>a11 a12 a13 a14 a15 a16 a17 a18 a19 a20<br>a21 a22 a23 a24 a25 a26 a27 a28<br>b1 b2 b3 b4 b5 b6 b7 b8 b9 b10<br>b11 b12 b13 b14 b15 b16 b17 b18 b19 b20<br>b21 b22 b23 b24 b25 b26 b27 b28 b29 b30<br>b31 ;<br><br>Missing = all (-1234) ;<br>Usev = lang<br>a2 a4 a7 a8 a9 a11–a28 ;<br>!a1 a2 a3 a4 a5 a6 a7 a8 a9 a10<br><br>Categorical =<br>a2 a4 a7 a8 a9 a11–a28 ;<br><br>!! single-group CFA<br>!Useobs = lang eq 0 ;<br><br>!! multiple-group CFA<br>!Grouping = lang (0=EL 1=ES) ;<br><br>Analysis:<br>BOOTSTRAP = 5000 ;	Model:<br>gen by<br>a2* a4 a7 a8 a9<br>a11 a12@1 a13 a14 a15 a16 a17 a18 a19 a20<br>a21 a22 a23 a24 a25 a26 a27 a28 ;<br><br>!pos by a1* a3 a5 a6 a10 ;<br>!neg by a4* a11 a12 a13 ;<br>cog by a14* a15 a16@1 a17 a18 a19 ;<br>lon by a8@1 a20@1 ;<br>soc by a21* a22 a23@1 a24 a25 a26 ;<br><br>!! orthogonality<br>gen with cog@0 lon@0 soc@0 ;<br>!gen with pos@0 neg@0 cog@0 lon@0 soc@0 ;<br>!pos with neg@0 cog@0 lon@0 soc@0 ;<br>!neg with cog@0 lon@0 soc@0 ;<br>cog with lon@0 soc@0 ;<br>lon with soc@0 ;<br><br>!! external covariate<br>gen cog lon soc on lang ;<br><br>Output:<br>standardized residual modindices ;<br>!standardized CINTERVAL (BCBOOTSTRAP);<br><br>Plot:<br>Type = Plot3 ;

Table S17 Bifactor CFA with language groups as external covariate (Model 3) in combined sample (n=1284) data for 23 **DEMQOL** items (standardised and unstandardised factor loadings with bootstrapped standard errors)

Qn	h^2	GEN			COG			LON			SOC		
		stdyx	unstd	*SE*	stdyx	unstd	*SE*	stdyx	unstd	*SE*	stdyx	unstd	*SE*
2	.42	.65	.93	.05									
4	.40	.63	.91	.04									
7	.42	.65	.93	.04									
8	.72	.54	.79	.04				.64	**1.00**	**			
9	.48	.70	1.00	.05									
11	.40	.63	.91	.04									
12	.49	.70	**1.00**	**									
13	.32	.56	.81	.04									
14	.62	.59	.85	.05	.52	.96	.08						
15	.58	.60	.85	.05	.48	.89	.08						
16	.57	.53	.75	.05	.54	**1.00**	**						
17	.74	.67	.96	.04	.54	1.00	.08						
18	.63	.69	1.00	.05	.39	.72	.08						
19	.65	.67	.96	.05	.45	.84	.10						
20	.77	.59	.86	.05				.64	**1.00**	**			
21	.62	.69	1.00	.05							.38	.66	.10
22	.70	.66	.96	.06							.50	.87	.11
23	.77	.65	.96	.05							.57	**1.00**	**
24	.63	.65	.94	.05							.45	.77	.07
25	.71	.71	1.04	.05							.44	.75	.08
26	.45	.55	.79	.06							.37	.64	.10
27	.57	.75	1.08	.05									
28	.40	.63	.90	.05									
ω_h		.89			.35			.48			.30		
lang		- .12	- .09^{ns}		.32	.18		- .46	- .30		- .72	- .42	

Model fit from non-bootstrapped results:
χ^2 = 1674.018 (df = 236), RMSEA = **.069** (90% CI = .066 - .072), CFI = **.925**
h^2: communalities
** unstandardised factor loading fixed at value of 1
ns: not statistically significant
ω_h: Omega hierarchical coefficient
lang: standardised (stdy) and unstandardized path coefficient for factor regressed on language (0=English, 1=Spanish)

Mplus syntax for 26 items in **DEMQOL-Proxy** Model 3 (Table S18)

```
Title:
combined-group bifactor CFA
for 26 DEMQOL-Proxy items

Data: File = p2croy1066.dat ;

Variable:
Names = lang f0 sev
a1 a2 a3 a4 a5 a6 a7 a8 a9 a10
a11 a12 a13 a14 a15 a16 a17 a18 a19 a20
a21 a22 a23 a24 a25 a26 a27 a28
b1 b2 b3 b4 b5 b6 b7 b8 b9 b10
b11 b12 b13 b14 b15 b16 b17 b18 b19 b20
b21 b22 b23 b24 b25 b26 b27 b28 b29 b30
b31 ;

Missing = all (-1234) ;
Usev = lang
     b2 b3     b5     b7     b9 b10
!b1 b2 b3 b4 b5 b6 b7 b8 b9 b10
       b12 b13 b14 b15 b16 b17 b18 b19 b20
!b11 b12 b13 b14 b15 b16 b17 b18 b19 b20
b21 - b31 ;

Categorical =
     b2 b3     b5     b7     b9 b10
     b12 b13 b14 b15 b16 b17 b18 b19 b20
b21 - b31 ;


!! single-group CFA
!Useobs = lang eq 0

!! multiple-group CFA
!Grouping = lang (0=EL 1=ES) ;

Analysis:
BOOTSTRAP = 5000  ;
```

```
Model:
gen by
      b2* b3   b5    b7    b9 b10
!b1* b2 b3 b4 b5 b6 b7 b8 b9 b10
b12 b13 b14 b15 b16 b17 b18 b19 b20
!b11 b12 b13 b14 b15 b16 b17 b18 b19 b20
b21 b22 b23 b24 b25 b26 b27@1 b28 b29 b30
b31 ;

!pos by b1* b4 b6 b8 b11 ;
neg by b2* b3 b5@1 b7 b9 b10 ;
app by b21@1 b22@1 ;
!fin by b23* b24 b25 ;
fin by b23@1 ;
fin by b24* b25 (eq12) ;
!soc by b27* b28 b29 b30 ;
cog by b12* b13 b14@1 b15 b16 b17 b18 b19 b20 ;

!! orthogonality
 gen with          neg@0 app@0 fin@0          cog@0 ;
!gen with pos@0 neg@0 app@0 fin@0 soc@0 cog@0 ;
!pos with neg@0 app@0 fin@0 soc@0 cog@0 ;
neg with app@0 fin@0  cog@0 ;
app with fin@0  cog@0 ;
fin with cog@0 ;
!soc with cog@0 ;

!! external covariate
gen neg app fin cog on lang ;

Output:
standardized residual modindices ;
!standardized CINTERVAL (BCBOOTSTRAP);

Plot:
Type = Plot3 ;
```

Table S18 Bifactor CFA with language groups as external covariate (Model 3) in combined sample (n=1404) data for 26 **DEMQOL-Proxy** items (standardised and unstandardised factor loadings with bootstrapped standard errors)

Qn	h^2	**GEN**			**NEG**			**APP**			**FIN**			**COG**		
		stdyx	unstd	*SE*	stdyx	unstd	*SE*	stdyx	unstd	*SE*	stdyx	unstd	*SE*	stdyx	unstd	*SE*
2	.54	.50	.68	*.05*	.57	**1.00**	**									
3	.59	.44	.60	*.05*	.66	1.17	*.09*									
5	.51	.44	.60	*.05*	.59	1.05	*.07*									
7	.55	.57	.78	*.05*	.50	.88	*.07*									
9	.33	.29	.40	*.05*	.51	.91	*.09*									
10	.46	.49	.67	*.05*	.50	.88	*.08*									
12	.63	.50	.69	*.05*										.65	**1.00**	**
13	.48	.52	.71	*.06*										.50	.75	*.07*
14	.84	.56	.79	*.04*										.76	1.18	*.05*
15	.63	.57	.79	*.05*										.59	.90	*.07*
16	.51	.61	.84	*.06*										.41	.62	*.08*
17	.61	.62	.85	*.05*										.52	.78	*.07*
18	.67	.67	.92	*.05*										.52	.78	*.08*
19	.60	.65	.89	*.05*										.47	.71	*.08*
20	.50	.64	.87	*.05*										.35	.52	*.09*
21	.90	.49	.75	*.05*				.77	**1.00**	**						
22	.92	.52	.79	*.05*				.77	**1.00**	**						
23	.64	.61	.83	*.05*							.52	**1.00**	**			
24	.83	.60	.82	*.05*							.68	1.32	*.10*			
25	.80	.58	.79	*.05*							.68	1.32	*.10*			
26	.47	.68	.93	*.04*												
27	.54	.73	**1.00**	**												
28	.41	.64	.87	*.04*												
29	.50	.71	.96	*.04*												
30	.53	.73	.99	*.04*												
31	.34	.59	.80	*.04*												
ω_h		.89			.50			.67			.49			.42		
lang		- .33	- .24		.70	.40		- 1.01	- .87		- .15	- .08^{ns}		.93	.62	

Model fit from non-bootstrapped results:

χ^2 = 2051.211 (df = 302), RMSEA = **.064** (90% CI = .062 - .067), CFI = **.935**

h^2: communalities

** unstandardised factor loading fixed at value of 1

ns: not statistically significant

ω_h: Omega hierarchical coefficient

lang: standardised (stdy) and unstandardized path coefficient for factor regressed on language (0=English, 1=Spanish)

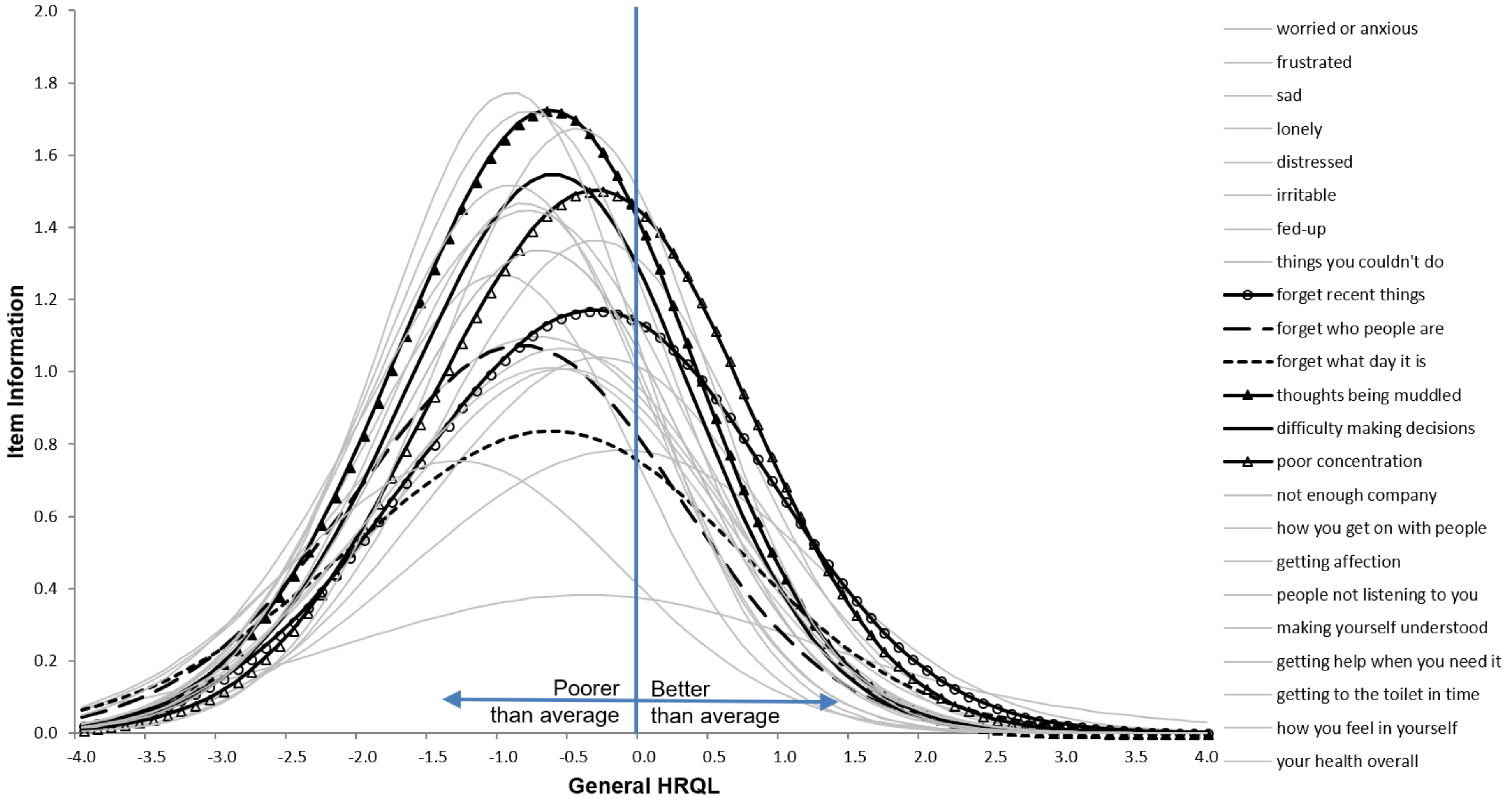

Figure 2 Item information curves of 6 “worry about cognition” items for self-report HRQL (DEMQOL Model 3)
Reported in main paper. Reproduced here for ease of comparison.

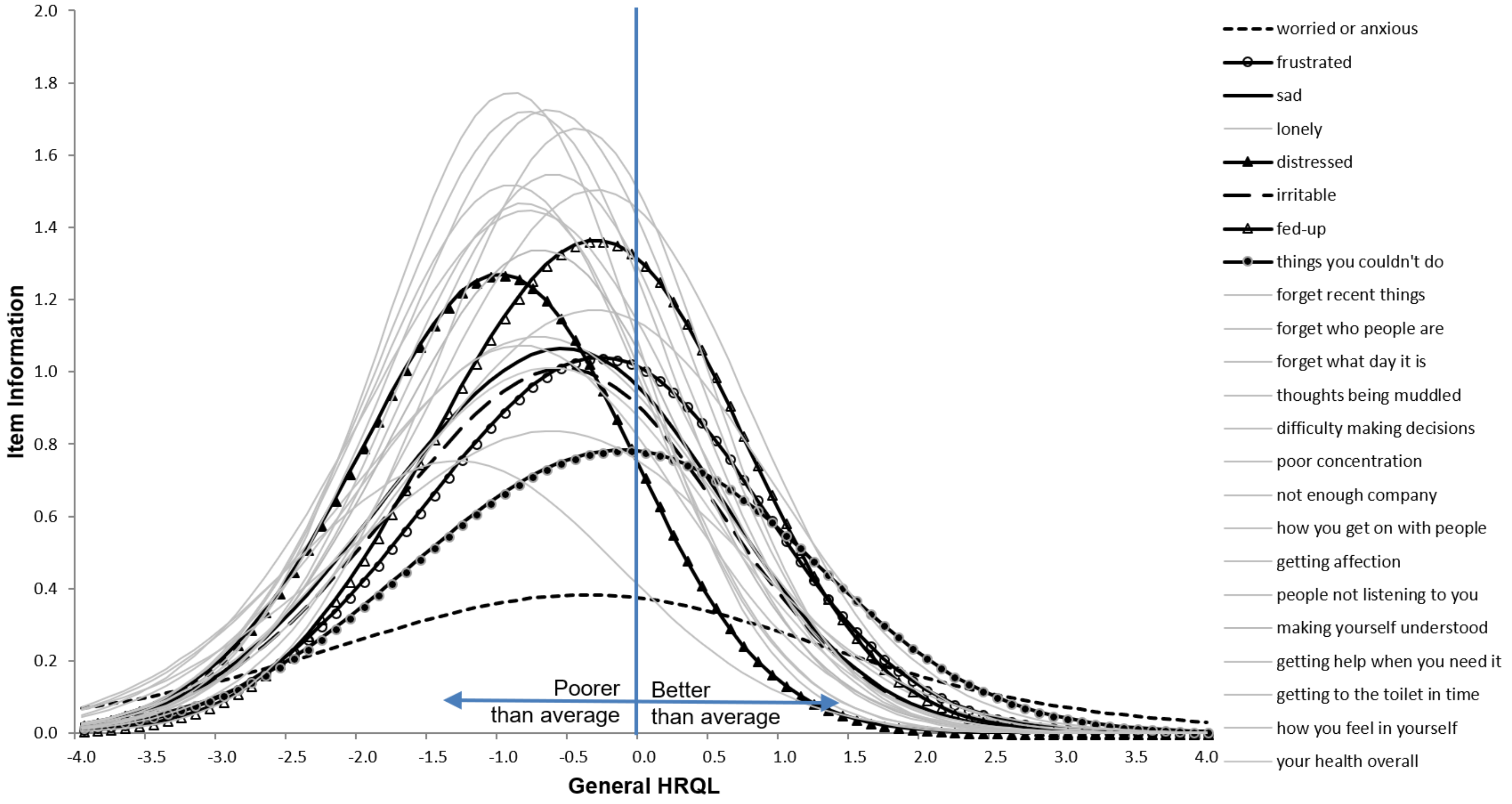

Figure S2a Item information curves of 7 "negative emotion" items for self-report HRQL (DEMQOL Model 3)

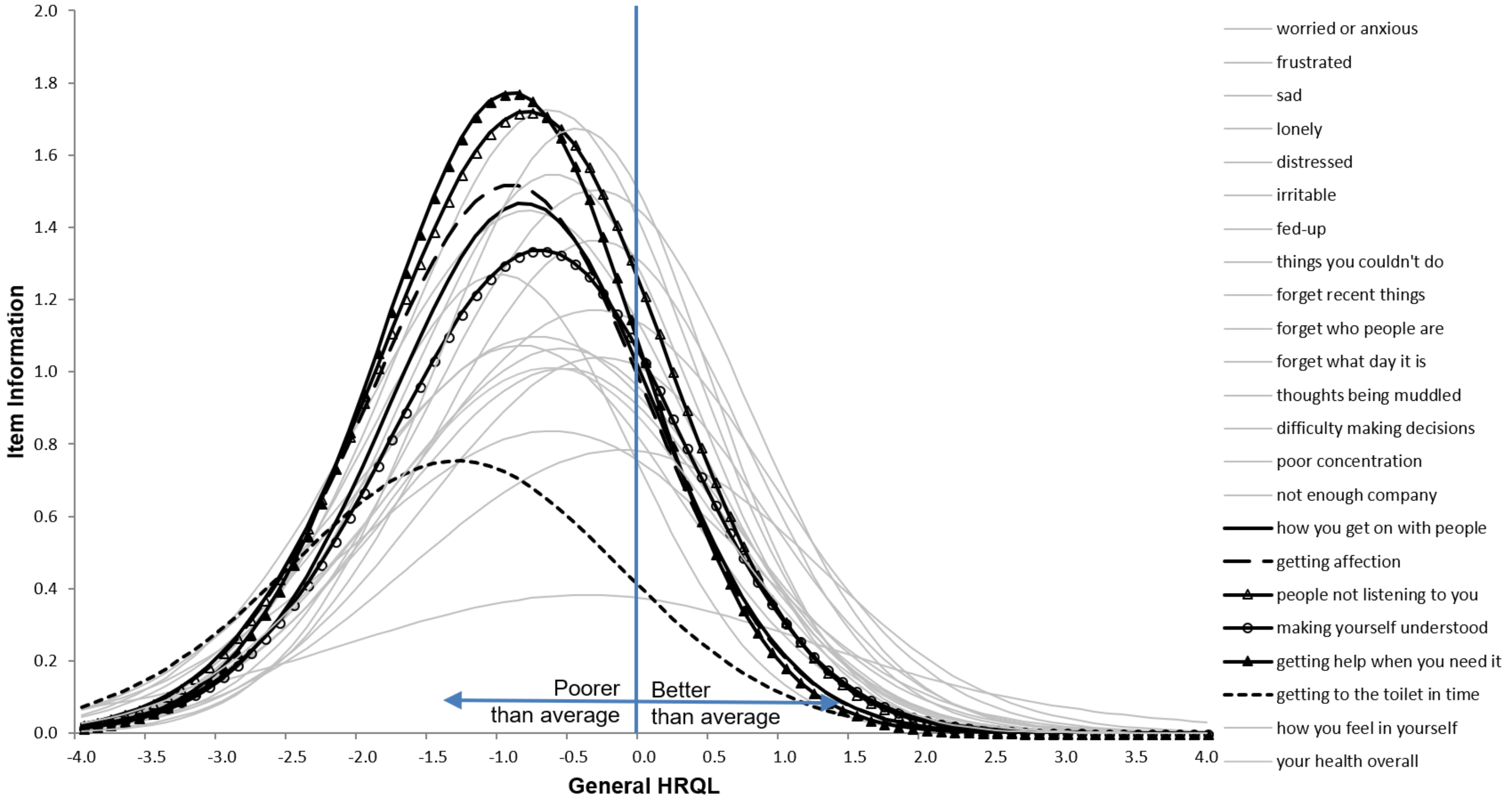

Figure S2b Item information curves of 6 “worry about social relationship” items for self-report HRQL (DEMQOL Model 3)

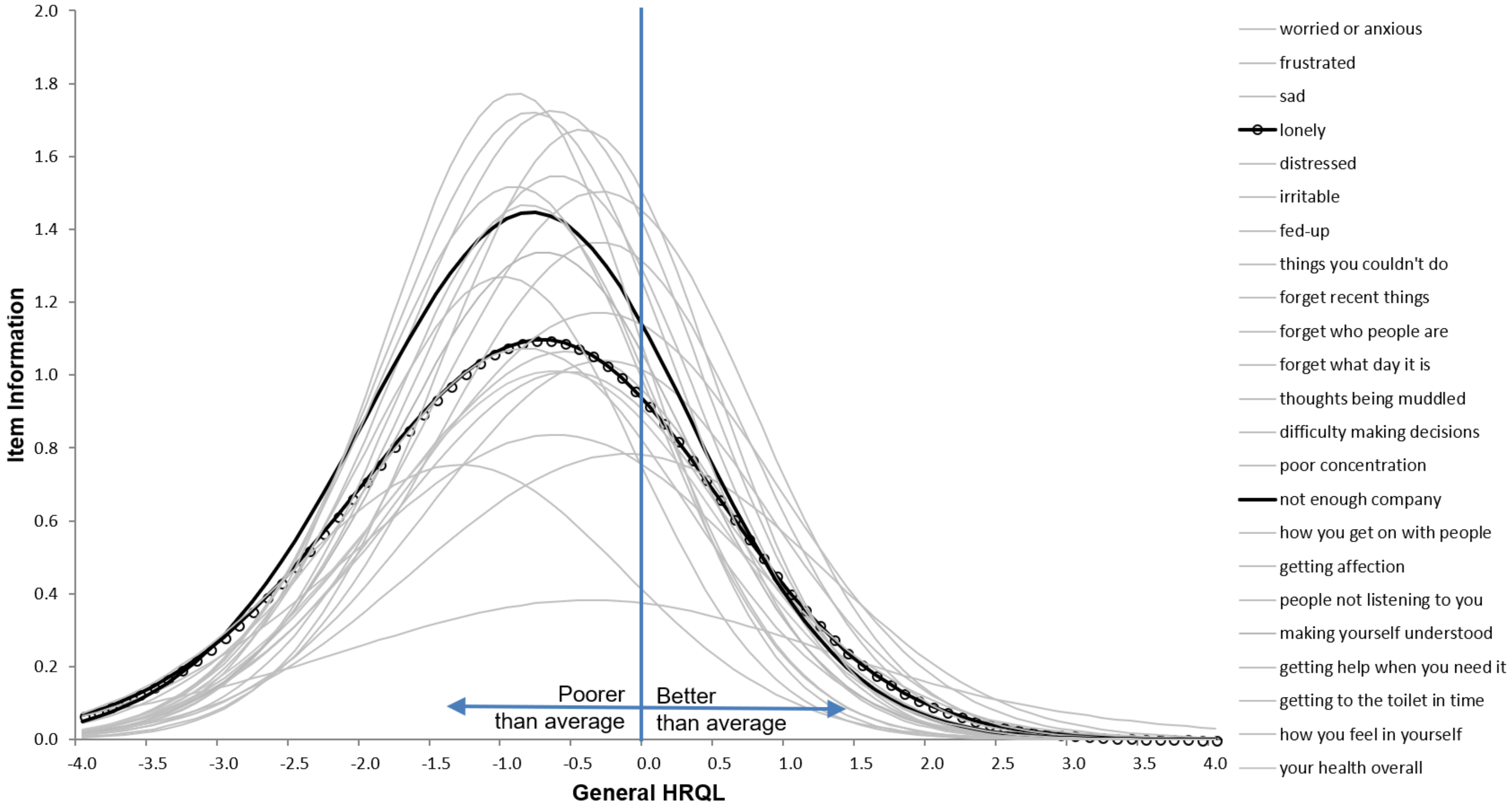

Figure S2c Item information curves of 2 “loneliness” items for self-report HRQL (DEMQOL Model 3)

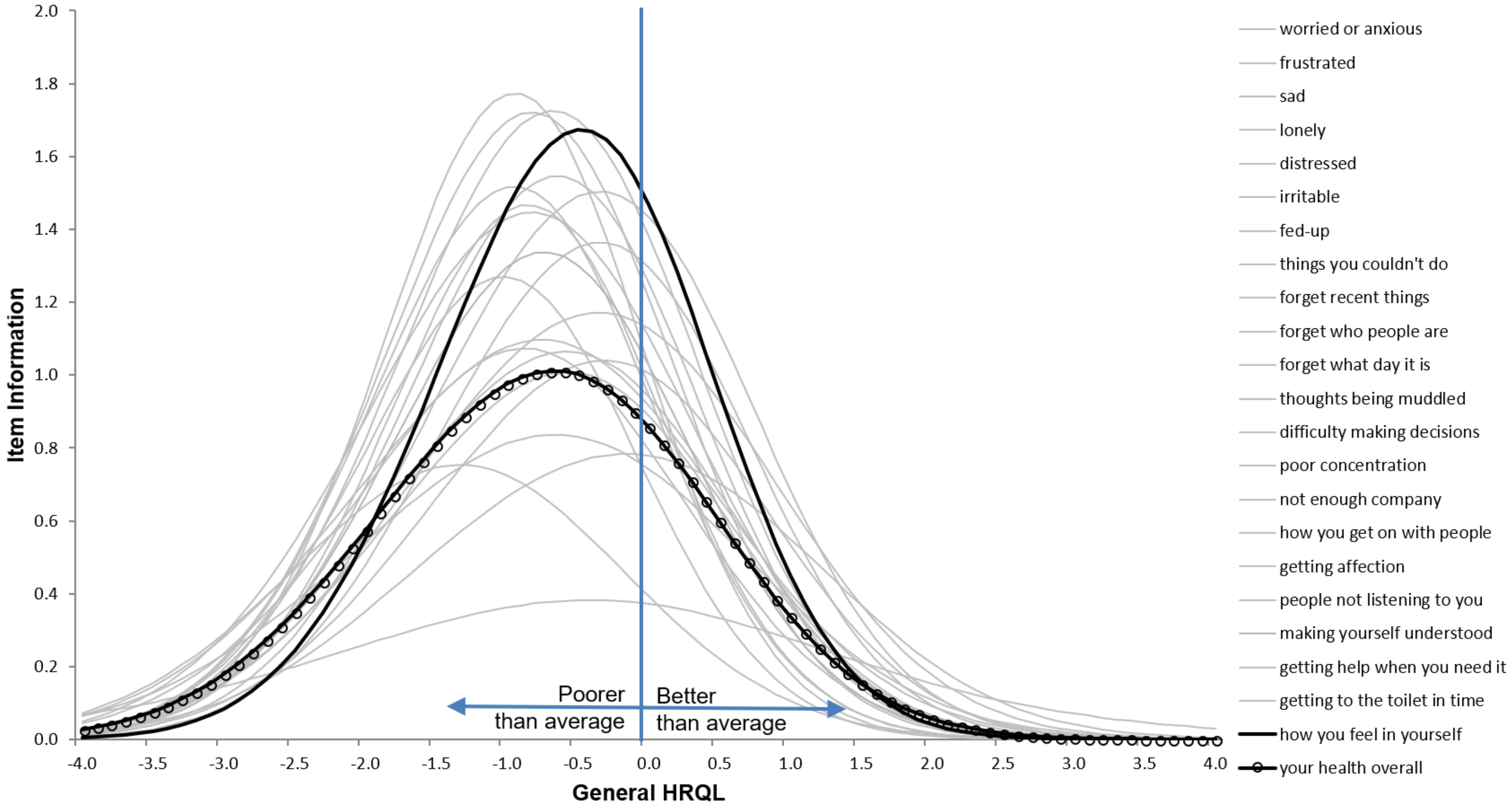

Figure S2d Item information curves of 2 items that loaded only on general HRQL for self-report HRQL (DEMQOL Model 3)

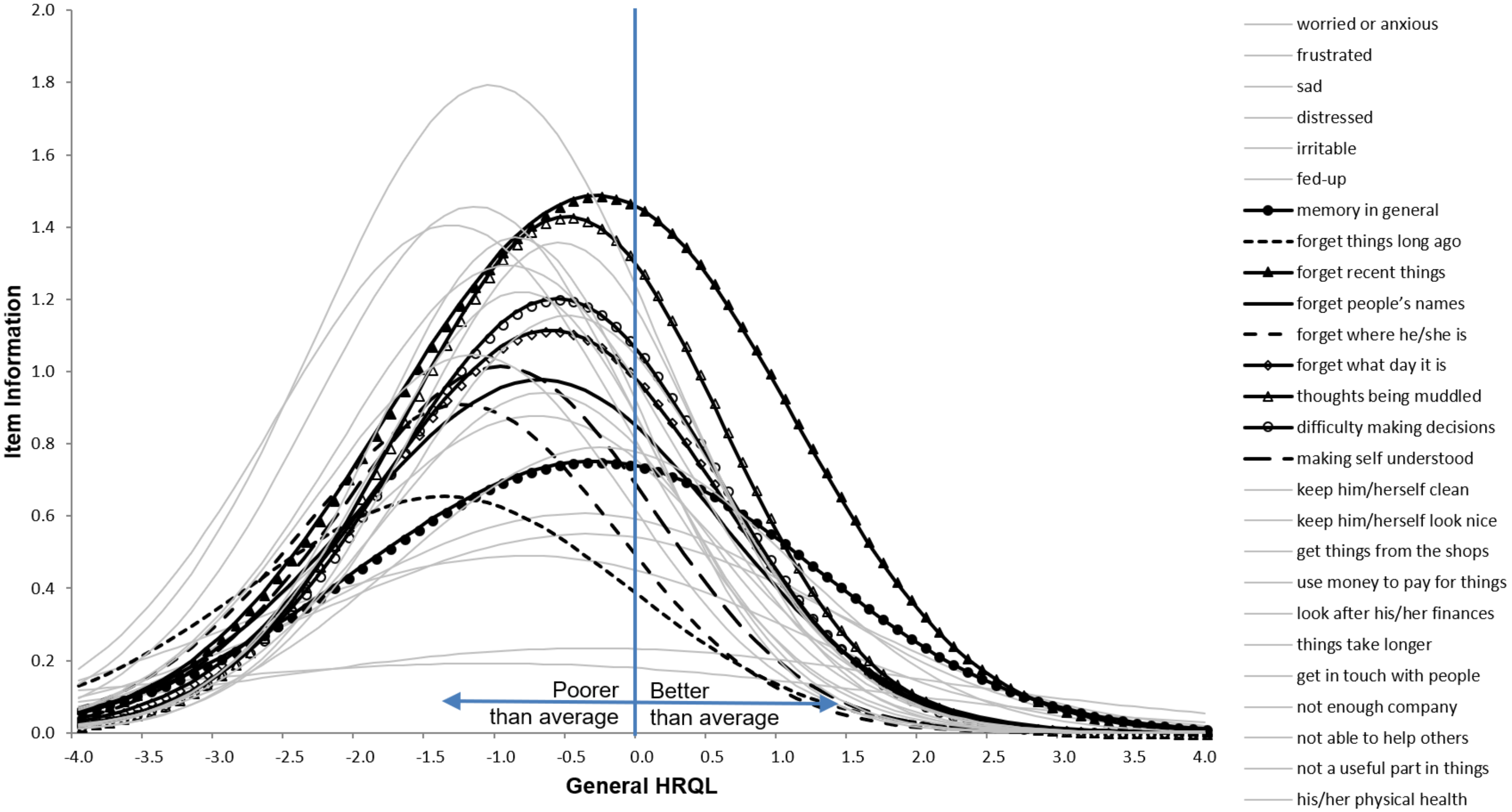

Figure 3 Item information curves of 9 "worry about cognition" items for informant-report HRQL (DEMQOL-Proxy Model 3)
Reported in main paper. Reproduced here for ease of comparison.

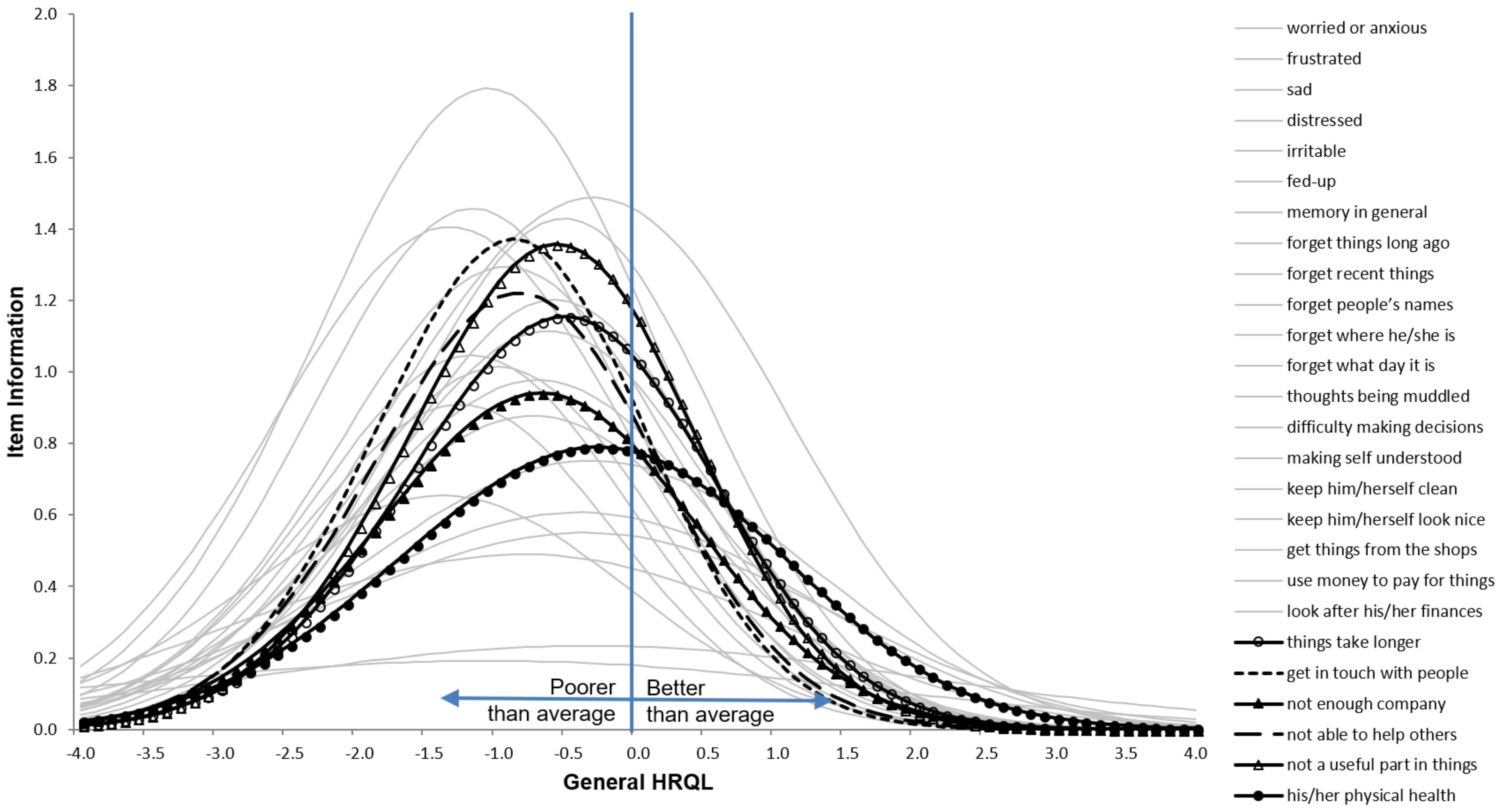

Figure S3a Item information curves of 4 "worry about social relationship" and 2 general HRQL items for informant-report HRQL (DEMQOL-Proxy Model 3)

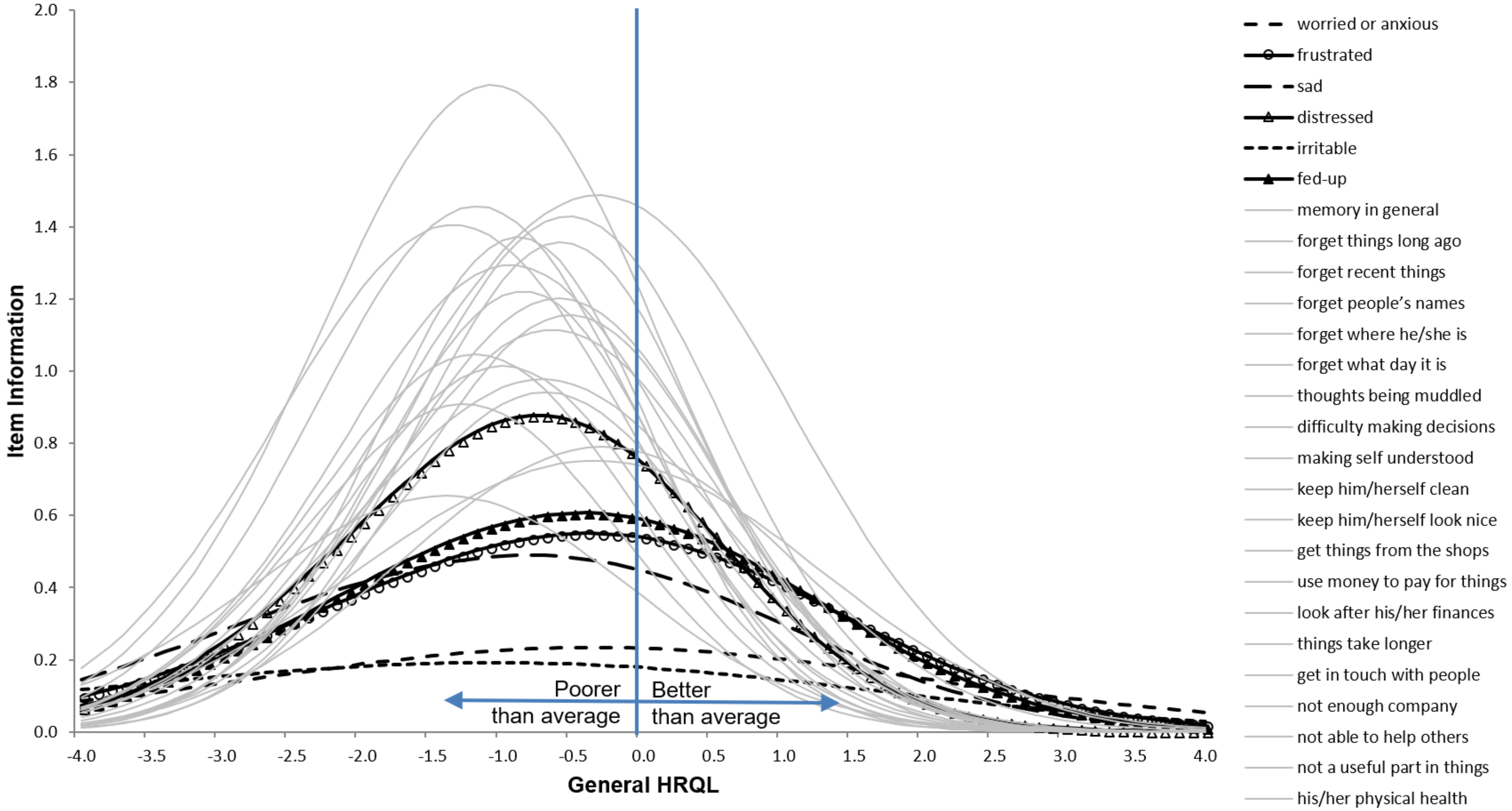

Figure S3b Item information curves of 6 “negative emotion” items for informant-report HRQL (DEMQOL-Proxy Model 3)

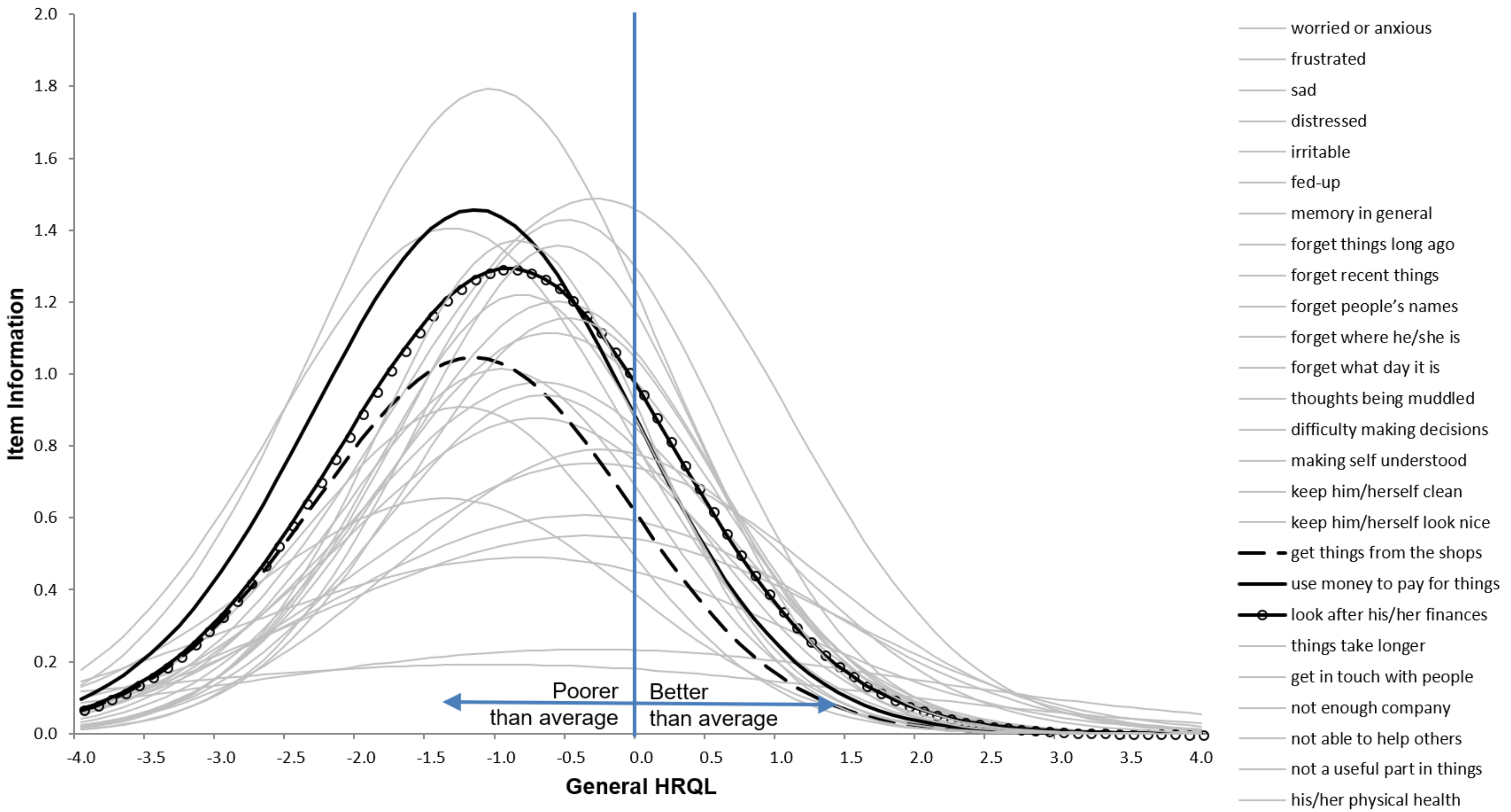

Figure S3c Item information curves of 3 "worry about finance-related tasks" items for informant-report HRQL (DEMQOL-Proxy Model 3)

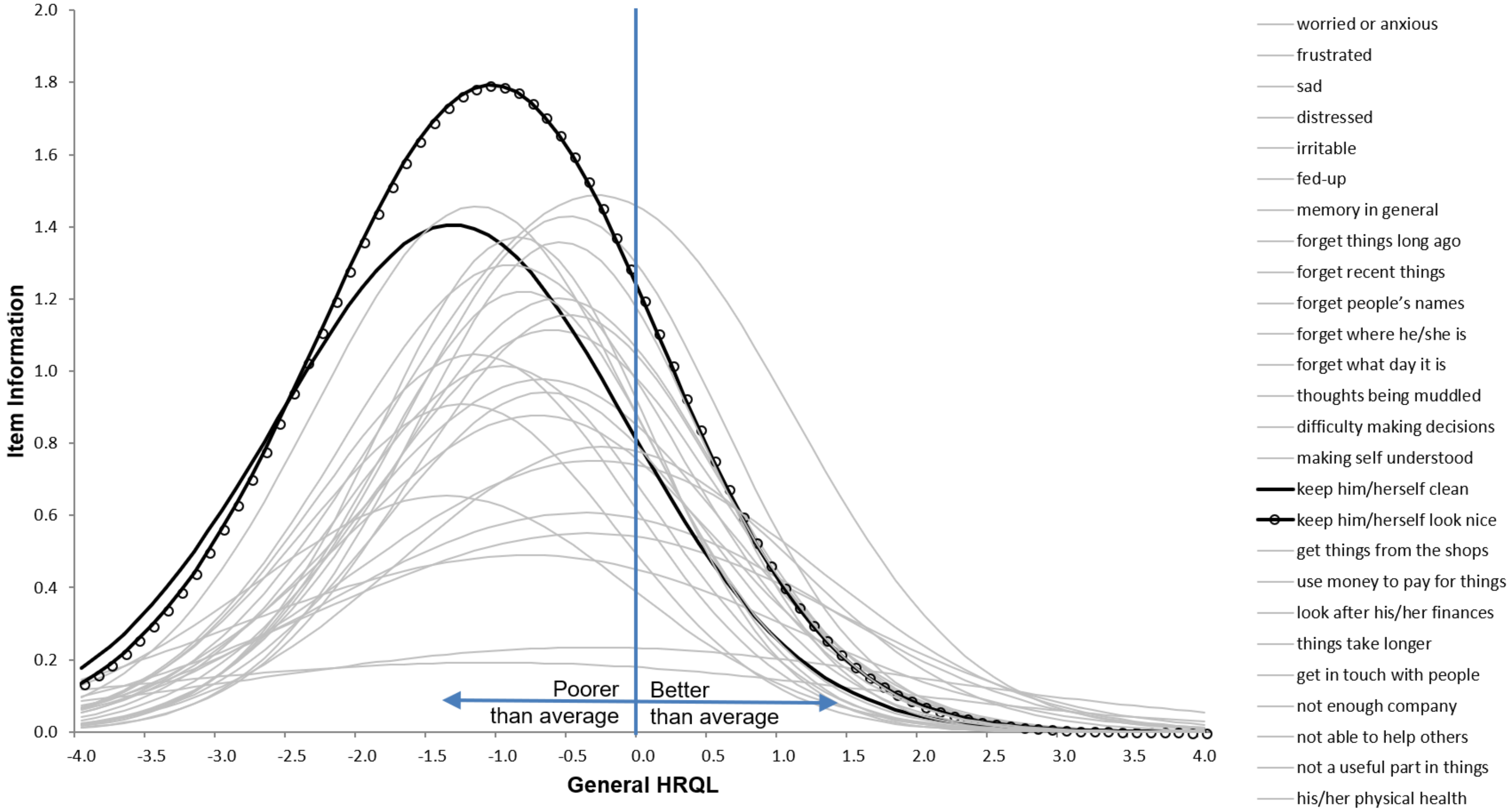

Figure S3d Item information curves of 2 “worry about physical appearance” items for informant-report HRQL (DEMQOL-Proxy Model 3)
